# First Report on the Acrobat Ant *Crematogaster scutellaris* Storing Live Aphids in Its Oak-Gall Nests

**DOI:** 10.3390/insects12020108

**Published:** 2021-01-27

**Authors:** Daniele Giannetti, Mauro Mandrioli, Enrico Schifani, Cristina Castracani, Fiorenza A. Spotti, Alessandra Mori, Donato A. Grasso

**Affiliations:** 1 Department of Chemistry, Life Sciences & Environmental Sustainability, University of Parma, Parco Area delle Scienze, 11/a, 43124 Parma, Italy; enrico.schifani@unipr.it (E.S.); cristina.castracani@unipr.it (C.C.); fiorenzaaugusta.spotti@unipr.it (F.A.S.); alessandra.mori@unipr.it (A.M.); donatoantonio.grasso@unipr.it (D.A.G.); 2 Dipartimento di Scienze della Vita (DSV), University of Modena e Reggio Emilia, 41125 Modena, Italy; mauro.mandrioli@unimore.it

**Keywords:** ant–plant interactions, ant-aphid relationships, mutualism, oak-gall secondary fauna, multitrophic interactions, ant behavior

## Abstract

**Simple Summary:**

Galls represent an amazing microcosm which contains a variety of multiple interactions among different actors, and therefore, offers the opportunity to observe and investigate phenomena belonging to different areas of biology: from the development process, connected to the interaction between the galligenous agent and the host plant, to the moment of their colonization by different species, since some ants may provide defense against pathogens, certain phytophagous insects or favor mutualists. In the present work we describe some aspects of oak-gall colonization by different ant species, highlighting how the gall’s height on the plant influences ant colonization and how different ant species produce different nest architectures. The most relevant aspect, however, is the discovery of a novel ant-aphid relationship: the transport of living aphids into oak-gall nests. We found no evidence of immediate predation of these aphids inside the galls, so they are likely stored to overwinter due to a mutualistic relationship and/or serve as food storage. This is not only an interesting report on the mutualisms involving ants and their insect partners, but it may also have important consequences on the aphids’ phenology with the host plants. Once more, ants show their relevant impact on multitrophic interactions and ecosystem dynamics.

**Abstract:**

This study provides new data about the role of ants in mutualistic interactions with aphids mediated by galls. We focused our investigation on galls induced by the cynipid *Andricus kollari* by conducting a survey and a subsequent experiment in an Italian oak forest. The ants *Crematogaster scutellaris*, *Colobopsis truncata* and *Temnothorax italicus* frequently used the galls as nests: *Crematogaster scutellaris* occupied galls which were located higher on the oak trees, while *C. truncata* and *T. italicus* were located at lower positions. In addition, galls occupied by *C. scutellaris* showed varied internal architecture in relation to the colony composition. Importantly, field surveys revealed for the first time that *C. scutellaris* nest galls also contained live individuals of the non-galligenous aphid *Panaphis juglandis*. Field experiments suggested that the ants actively seek, collect and stock live aphids. No signs of predation and injuries were detected on the stored aphids, which were probably kept for safe overwintering, though we cannot exclude a possible occasional use as food. This report reveals a possible novel relationship which could have important consequences on the phenology and presence of aphids on the host plant.

## 1. Introduction

Galls are neoformed structures developed on plants in response to a parasite attack, called “inductor,” which is most usually an insect. As such, galls are regarded as a product of ecosystem engineering. From the individual gall-inductor perspective, the changes in growth and development that are elicited in the plant are unique, for the most part being species-specific [1,2,3]. However, after the hatch and departure of the inductor’s offspring, galls may be occupied by secondary fauna, in particular by ants that can start a close relationship with the host plant, which in turn can gain important advantages from the ants’ presence by gaining protection from phytophagous insects and pathogens [4].

Among insects, ants have a special place due to their abundance, social organization, and amazing adaptation to different lifestyles, competition, and resource exploitation, which also leads to several symbiotic interactions [5,6,7,8,9,10]. Ant-aphids associations are a textbook example among symbioses involving ants, representing special alliance described in terms of nutrition/protection exchange. Specifically, ants offer aphids protection against enemies and pathogens and in return are provided with honeydew [11]. Besides being extremely rich in sugar, this honeydew may be additionally enriched with amino acids so as to be more attractive to ants. Hence, the great abundance of aphids from spring to summer represents a precious source of nutrients so that it is advantageous for the ants to tend and protect them from both parasitoids/predators and other possible environmental insults [11,12,13,14]. Interspecific cooperation represents a leading force in evolution [15]. Mutualisms have, therefore, received increasing attention not only due to their ecological implications, but also because their study represents an important interpretative tool in evolutionary and developmental biology, genetics, immunology and physiology [16,17,18].

The strength of these mutualisms is highly variable: it may be obligate or facultative and may have developed independently several times [13,19,20,21]. In some cases, mutualistic ants may simultaneously protect and prey on the aphids they tend [19,22]. On the other hand, ants can even be induced to tend non-myrmecophilous species if there is no other source of carbohydrates available [20]. In case of competition between two distinct species of aphids, ants may protect their mutualistic partner species by removing the competitor [23]. Ant services offered to aphids may take several different forms, in addition to direct protection against predators. Some ant species bring adult aphids into their nests during wintertime to ensure aphid survival and abundance in the following spring, a phenomenon frequently reported for aphids of the tribe Fordini [12,13]. Other ant species store aphid eggs within their nest in autumn in order to favor aphid survival as eggs during the winter (overwintering). At spring, aphids hatch in the nest and ants move the newly hatched parthenogenetic aphids back to the plants to farm them in order to have a larger amount of honeydew as a consequence of the aphid population’s growth [24]. This kind of herding is reported in several trophobiosis cases involving aphids and other honeydew-producing hemipterans [25]. Furthermore, as in the case of *Lasius flavus* (Fabricius, 1782), some ant species exhibit a sort of clonal preference for aphids so that their “husbandry” is characterized by low aphid “livestock” diversity per colony, especially at the nest-chamber level, and ant queens leaving to start a new colony take an aphid egg to found a new herd of underground aphids in their new colony [26]. However, ant-aphid mutualism is much more complex than a mere nutritional/protection exchange between two partners and may have special ecological relevance. Various studies have revealed that mutualisms are embedded in multitrophic scenarios whose top-down/bottom-up effects are often underscored [19,27]. Selective pressures shaping (or deriving from) these interactions could be quite different in the different ecological contexts and may significantly affect morpho-functional and life-history traits, as well as population dynamics of interacting parties [12,25,28,29]. 

Overwinter survival is a significant challenge to aphids, which have adapted different strategies to deal with it. Most aphids overwinter as eggs, which may reach super-cooling points of −42 °C [30], that are laid on woody host plants, as is the case for the cherry aphid *Rhopalosiphum padi* (Linnaeus, 1758) [31,32], or in bark crevices, or even in lichens growing on the bark, as reported for the sycamore aphid *Drepanosiphum platanoidis* (Schrank, 1801) [33]. The egg-laying stage, in which both parthenogenesis and sexual reproduction coexist (holocyclic), is a part of a complex life cycle adopted by most aphids to pass the winter: this stage consists of one generation of sexual morphs (sexuals) and several generations in which only parthenogenetic females are produced [34]. Interestingly, some aphids have lost the amphigonic (sexual reproduction through the participation of two gametes) part of their life cycle, which is called anholocyclic. Some species are entirely anholocyclic (e.g., *Tuberolachnus salignus* (Gmelin, 1790), *Pineus boerneri* Annand, 1928, *Myzus ascalonicus* (Doncaster, 1946)), or anholocyclic in warmer climates and holocyclic in cold temperate regions (such as *Myzus persicae* (Sulzer, 1776)). In this last case, aphids pass the winter in an active stage, either as an adult or immature nymph, since, despite their soft bodies and fragile appearance, aphids have a rather low super-cooling point that allows aphids to survive temperatures as low as −26 °C [35]. A potential advantage of using an active overwintering stage and not an egg is that if they survive the winter, they can start reproducing sooner, particularly if they are a host-alternating species, where the aphids hatching from eggs must spend time developing and reproducing on the primary woody host before being able to migrate to the secondary hosts, where they start the parthenogenetic reproduction. This same advantage of earlier reproduction also applies, to a lesser extent, to those holocyclic aphids living on herbaceous plants, although the temporal advantage is not as great [36]. Furthermore, even if eggs are cold resistant, aphid egg mortality is typically around 70–80%, and generally, the longer the winter, the greater the mortality since the length of the winter (and not just the temperature) also determines how many aphid eggs will survive [31].

In the present study, we show that interaction between ants and aphids can be mediated by galls not produced by the aphids themselves but by other insects, and in particular, that ant-colonized galls may host aphids during overwintering.

Here, we report the first investigation on *Andricus kollari* (Hartig, 1843) gall colonization by ants. In particular, our study aimed to:

- Document which ant species colonized galls, what kind of colony composition characterized the gall-colonizing colonies (whether queens, workers or immature stages are present), and whether the galls’ height was a significant factor for colonization by different ant species.
- Document and quantify the presence of aphid species, suggested by some preliminary observation, and its relation to ant presence.
- Document how the presence of gall-colonizing ants or aphids influenced the galls’ internal architecture.

To pursue these aims, we operated by first conducting a survey collecting galls naturally grown on oak trees, and then through a field experiment in which we artificially placed empty galls on oaks by attaching them to twigs’ buds and observed their colonization status one year later.

## 2. Materials and Methods

### 2.1. Study Area and Gall Selection

**Figure 1:**
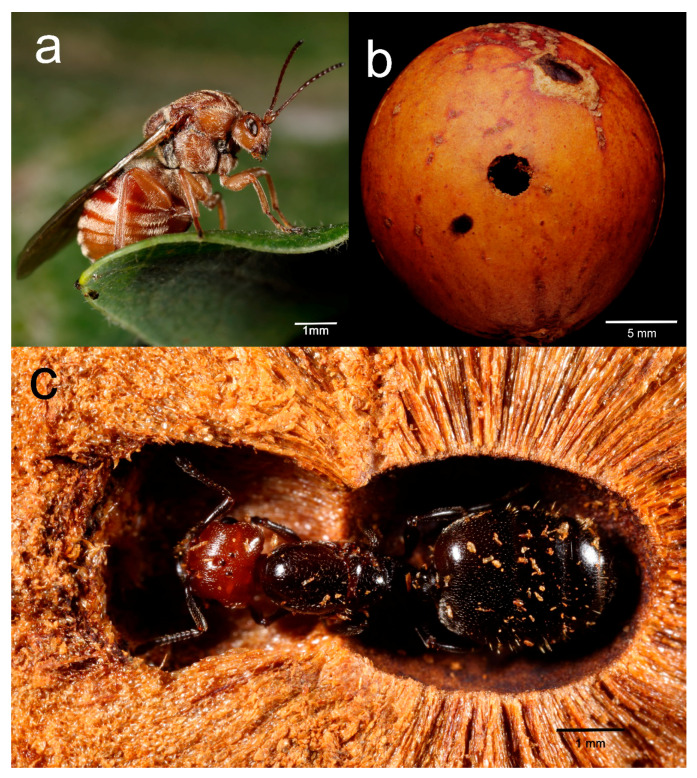
(**a**) *A. kollari* (Hymenoptera: Cynipidae) and the induced oak-gall (**b**); (**c**) a queen of *Crematogaster scutellaris* inside the gall of *A. kollari* during the first phase of gall colonization and colony founding.

The field survey and the experiment were conducted in Northern Tuscany (Italy) near the village of Fornoli (MS- 44°15′15.1″ N 9°58′01.8″ E). During the study, only galls induced by *A. kollari* (Hymenoptera: Cynipidae) (Figure 1) and located on *Quercus* spp. mature trees were selected. Galls were identified by their external morphology, characterized by smooth aspherical bright-brown structure, perhaps with a surface bump [1].

### 2.2. Survey

**Figure 2:**
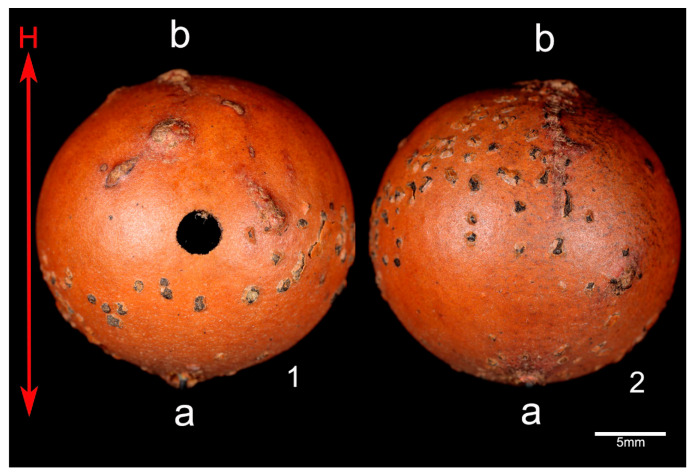
Galls used for the survey were divided into two halves (1, 2) according to the height line (H), keeping the hole produced by the emerging cynipid in the middle of one of the halves. The insertion point of the gall is also shown on the tree branch (a) and the opposite peak (b).

A total of 159 galls were collected from oak trees (*n* = 11) in September 2015. We selected only galls presenting a typical wasp’s hole that was already abandoned by the cynipid. For each of the collected galls, its height from the ground was measured by using a laser distance meter (Leica Disto D2). In the laboratory, all the galls were checked and those displaying physical damage, mold or signs of the presence of other arthropods (e.g., spiders or beetles, detected by the presence of silk or feces near the entrance hole) were excluded from further analyses. Each of the remaining galls was then cut into two halves tracing the height line keeping the gall’s hole approximately in the middle of one of the halves (Figure 2) as in [4]. The presence of ant queens (Q), workers (W), brood (b) or aphids (a) was checked. For all encountered aphids we assessed their condition, recording in particular: (a) eventual signs of biting or body fragments to verify the possible consumption or damage by the ants; (b) aphids’ movement in response to disturbance by the operator (using an entomological forceps for 30 s)—a proxy used to judge whether the aphids are alive. 

### 2.3. Field Experiment

A set of 177 ant-free galls was picked from mature oak trees in order to check whether ants are used to stocking aphids in their gall nests. These galls were selected among the ones which had not been occupied yet by secondary fauna as they lacked the exit hole of the cynipid. Each gall was then placed in perforated plastic boxes and kept under controlled lab conditions (22–24 °C, 50% RH). All galls were scanned daily for five months and the ones from which no *A. kollari* had hatched or which showed presence of mold or parasitoids were excluded from the subsequent experiments. 

**Figure 3:**
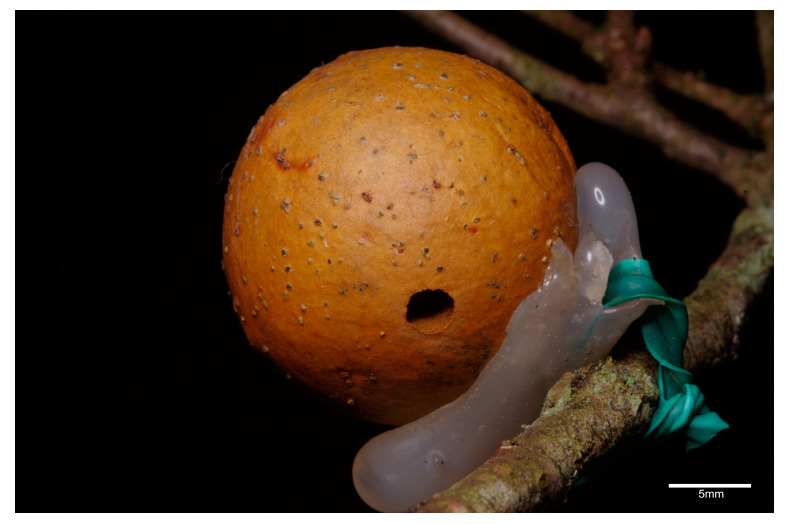
An experimental gall fixed with an artificial support method on the tree.

The remaining galls, from which wasps normally emerged (*n* = 100), were used for the field experiments, fixed on artificial supports (Figure 3) and placed back on the trees. A set of experimental galls (*n* = 50, ten per tree) was artificially placed on five trees of *Quercus* spp. at different heights (from 1 to 4.4 m: equally divided between the following heights: 1.5, 2, 2.5, 3, 3.5, 4, 4.4), so that all could be exposed to ant colonization. Another set of galls (*n* = 50) was used as control group from which ant presence was excluded. Aphid presence was detected in all the experimental trees by inspecting the branches. All encountered aphids belonged to one morphotype. In the case of the control group trees, before placing the control set, each tree was first checked to exclude the presence of pre-existing ant nests inside the trunk, and at least two aphid-colonized branches were detected (focal branches). Once the absence of ant nests was verified, each tree was softly beaten for 10 min with a wooden stick so as to induce any ant present on the tree to leave (for uniformity, the same treatment was used for the experimental trees, where ant access was freely allowed, see above).

This method had already been used in previous pilot trials and proved to be extremely effective in inducing any ants present on the tree to leave (Giannetti, unpublished data). After the treatment the tree was checked to definitely exclude the presence of any solitary ants and to ensure that aphids were still present on the focal branches. Later, the trees were isolated with adhesive strips (glue bands, Stocker art. 45118) at the base of the trunk to prevent any successive ant colonization [4,37]. The galls were left on the trees for one year before being retrieved (from September 2016 to October 2017, coinciding with the period of *Crematogaster scutellaris* (Olivier, 1792) nuptial flights and foundation of new colonies [38]). All galls were then cut into two halves (as in Figure 2) and the content was evaluated: ant species, colony composition (queens only; queen, workers and brood; workers only), and presence of aphids. The condition of each aphid was assessed by following the same procedure mentioned for the survey.

### 2.4. Determination of Aphid Species by DNA Barcoding

A molecular determination was performed for the aphid specimens collected in the galls. In particular, DNA extraction was performed by whole genome DNA extraction from single aphids by using the SW Genomic DNA extraction kit (Promega) following the manufacturer’s instructions. The amplification of a 700 bp long fragment of the gene coding for the cytochrome C oxydase I (COI) was performed using the LepF (5′-ATTCAACCAATCATAAAGATATTGG-3′) (forward) and LepR (5′-TAAACTTCTGGATGTCCAAAAAATCA-3′) (reverse) primers, based on the procedure reported by [39]. The PCR conditions and processes were as follows: an initial 5 min denaturation step at 95 °C, followed by 35 cycles consisting of 20 s at 94 °C, 30 s at 50 °C, 2 min at 72 °C, and finally a 7 min extension step at 72 °C. 

Sanger sequencing of the amplified fragments was performed at BMR Genomics (Padua, Italy), and the COI sequences obtained were aligned by using the identification tool freely available at the Barcoding of Life Database (BOLD) (http://www.boldsystems.org/index.php/IDS_OpenIdEngine). 

Sequence alignment was performed at EMBL-EBI with CLUSTAL Omega (https://www.ebi.ac.uk/Tools/msa/clustalo/), whereas alignment editing was carried out with *MView* (https://www.ebi.ac.uk/Tools/services/web/toolform.ebi?tool=mview&sequence).

### 2.5. Identification of Ant Species 

Specimens were examined under a ZEISS Stemi 508 stereoscopic microscope (5–200× magnification range) with the support of an Axiocam Erc 5s and ZEISS ZEN core software used to take morphometric measurements. Ants were identified following specific taxonomic keys [38,40].

### 2.6. Inner Architecture: Excavation Volume and 2D Analysis 

Both analyses were performed on the same galls to evaluate the proportion of material removed by the ants, and the structure and size of the chamber where the aphid was placed. The galls used for the analysis were selected as subsets of the ones sampled during the field experiment. In order to analyze the proportion of material removed by the ants according to species colonization and colony composition, we selected 20 galls (seven that were empty, two with a queen of *C. truncata* (Spinola, 1808) only, four with a queen of *C. scutellaris* only and aphids, seven with a queen, workers, brood of *C. scutellaris* and aphids). 

The excavation volume of galls was assessed using silica micro-gel characterized by an average diameter (Ø = 0.055 mm). An amount of micro-gel was poured inside each half of the galls to fill the chambers excavated by the ants. Finally, the excavation volume was determined by the weight of the micro-gel needed to fill the chambers compared to the known weight of 0.1 mL micro-gel (BEL Analytical Balances). 

In addition, the galls with aphids were measured by 2D excavation area analysis. In particular, we selected 11 galls of *C. scutellaris*: four hosting the ant queen only and aphids, and seven hosting the queen, workers, brood and aphids. 

A 2D excavation area image was obtained for each half of each gall by using the stereomicroscope Zeiss Stemi 508, the Axiocam Erc 5s, and a focus stacking technique [4]. The excavation area of the entire gall and of the aphid chamber was measured by using the Zeiss Zen core Software, and average values for each gall were obtained from both the halves.

### 2.7. Statistical Analyses

Statistical analyses were performed by using IBM statistical software SPSS 20.0 for Windows package, as well as R (v4.0.2) and R Studio (v1.3.1056) (R Core Team 2020).

#### 2.7.1. Survey

The differences in gall selection by different ant species depending on the gall’s height on the tree were assessed through a linear mixed model using the R function lmer() from the package lme4 [41]. The gall’s height from the ground (m) was set as the dependent (continuous) variable, while the ant species present was set as the independent variable, and the tree hosting the galls was added as random factor. Four categories were created for *C. scutellaris* only, the most abundant ant species present in the galls, in order to facilitate statistical analyses depending on ant colony composition and the presence of aphids: 1: galls with queens only (Q); 2: galls with a queen and aphids (Qa); 3: galls with a queen, workers, brood and aphids (Qwba); 4: galls with queen, workers and brood (Qwb). Tests were computed by using the lmer() function again, the gall’s height was used as the dependent variable, and the colony composition or presence/absence of aphids were used as the independent variables, while the tree remained a random factor. In all the above-mentioned tests, pairwise comparisons were computed by using the R function lsmeans() from the lsmeans package [42] whenever statistically significant differences were detected.

#### 2.7.2. Field Experiment

According to data distribution, either a one-way ANOVA or a Kurskal–Wallis test were used to investigate differences on gall selection by ant species, using the galls’ height as the variable and the species as the factor.

#### 2.7.3. Excavation Volume Analysis

The excavation volume of the ant chamber and aphid chamber was tested with a one-way ANOVA. Four categories were created to facilitate statistical analyses and to separate the possible role of queens’ presence from workers presence across the spectrum of *C. scutellaris* colony compositions. Each gall was classified as either empty, or according to the four categories of ant colony composition and the presence of aphids; 1: galls with queen of *C. truncata*; 2: galls with queen of *C. scutellaris* and aphids (Qa); 3: galls with queen, workers, brood of *C. scutellaris* and aphids (Qwba); 4: galls with workers of *C. scutellaris* only. 

#### 2.7.4. Two-Dimensional Analysis (Nest Architecture)

The effect of aphid presence on the excavation area (mm^2^) was tested with a one-way ANOVA test and Tukey’s post hoc tests were performed when statistically significant differences were detected. 

## 3. Results

### 3.1. Survey

After the preliminary scan, the galls were classified according to their occupants (*n* = 130): 43 galls had been colonized by ants (29 by *C. scutellaris*, 9 by *C. truncata*, 5 by *Temnothorax italicus* (Consani, 1952)), 36 galls were empty and 51 had been colonized by spiders (data are available in Table S4). 

The galls colonized by *C. truncata* were occupied by a queen alone. Quite differently, each gall colonized by *T. italicus* was occupied by a complete colony, made up of a queen, workers and brood. *C. scutellaris* exhibited the most heterogeneous colony composition: 16 galls hosted a queen alone; 3 galls hosted a queen and aphids; 8 galls hosted a queen, workers, brood and aphids; 2 galls were occupied by a queen, workers and brood but no aphids. Therefore, the presence of aphids was never detected in galls not colonized by ants, as it was only recorded inside *C. scutellaris* gall nests (further information below). Aphids were less numerous in galls occupied by single *C. scutellaris* queens (min–max: 3–5) than in the ones containing workers and brood (min–max: 6–11) (one-way ANOVA: F_1,9_ = 8.5; *p* = 0.017). No flying aphids were found.

**Figure 4:**
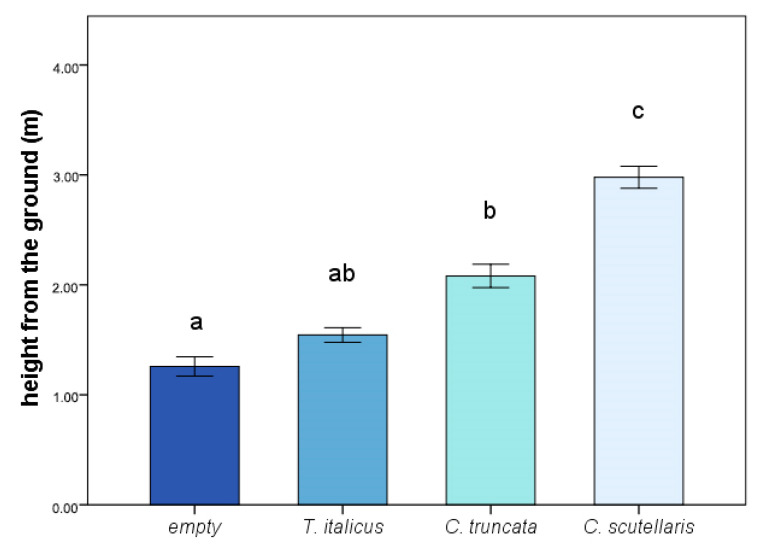
Gall occupancy according to position on the tree (height from the ground) and ant species. SE interval is shown for each bar. The bars with the same letter are not statistically different (one-way ANOVA and Tukey’s post-hoc tests).

Significant differences were recorded when considering the relationships between gall height from the ground and colonizing ant species (*n* = 79; F_3,75_ = 65.16, *p* < 0.001). Pairwise comparisons highlighted that the *C. scutellaris* gall’s height was statistically different from all the other categories (showing the highest values, Mean ± SE; 2.97 ± 0.09 m; 0.002 < *p* < 0.001). The height of the empty galls (showing the lowest values, Mean ± SE; 1.2 ± 0.08 m) and *C. truncata* galls (Mean ± SE; 2.08 ± 0.10 m) were significantly different one from the other (*p* = 0.003), but no significant difference was found when each was compared to *T. italicus* (Mean ± SE; 1.5 ± 0.06 m; 0.63 < *p* < 0.25) (Figure 4).

When focusing on galls colonized by *C. scutellaris* (*n* = 29), the analysis showed no statistical difference for the gall height from the ground in relation to the ant colony composition (Mean ± SE; Q 2.8 ± 0.15 m; Qa 2.9 ± 0.29 m; Qwba 3.1 ± 0.09 m; Qwb 2.8 ± 0.52 m) (F_3,25_ = 1.09, *p* = 0.37). No statistical differences were found for the gall height from the ground in relation to the presence of aphids either (galls with and galls without aphids) (Mean ± SE: with aphids 3.11 ± 0.10 m; without aphids 2.89 ± 0.14 m; F_1,27_ = 0.11, *p* = 0.74). 

### 3.2. Field Experiment

**Figure 5:**
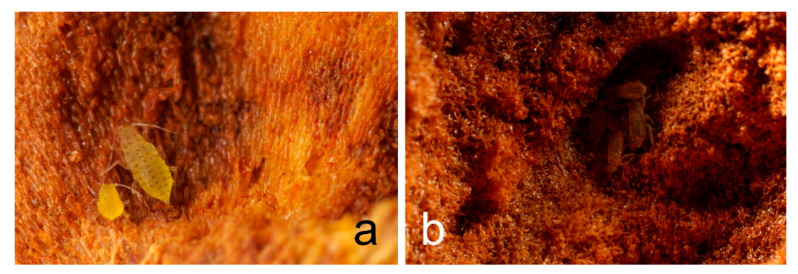
*Panaphis juglandis* (**a**,**b**) at different stages of development, still alive and with no signs of damage caused by ants inside a gall nest of *C. scutellaris*.

The analysis of the experimental galls (*n* = 50) placed on trees where ants were allowed revealed that 16 hosted ants (3 colonized by a queen of *C. truncata* alone, 13 by *C. scutellaris*), 12 were colonized by spiders and 22 were empty (data are available in S4). No control galls, placed on ant-excluded trees, had been colonized by ants or aphids or other arthropods. Here, as recorded in the field survey, we only found aphids in experimental galls colonized by *C. scutellaris* (Figure 5). Aphids were located in well-defined chambers inside the galls resulting from excavation by the ants, separate but connected to the main chamber hosting ants. No flying aphids were found. They showed no sign of evident damage and suddenly moved following disturbance (30 s) by the investigator. The colony composition in the experimental galls colonized by *C. scutellaris* (*n* = 13) was as follows: two galls with workers only (W); four galls with Queen and aphids (Qa); seven galls with a queen, workers, brood and aphids (Qwba). No preference for the gall height from the ground was found in relation to colony composition by *C. scutellaris* (Mean ± SE: W 3.0 ± 0.05; Qa 3.1 ± 0.26; Qwba 3.5 ± 0.25, One-Way ANOVA F_2,10_ = 0.77, *p* = 0.48). Aphids were less numerous in galls occupied by single *C. scutellaris* queens (min–max: 4–6) than in those also containing workers and brood (min–max: 7–18) (one-way ANOVA: F_1,9_ = 4.1, *p* = 0.073). No statistically significant differences were found between the height of galls colonized by *C. scutellaris* and empty galls (H^2^_3_ = 4.10, *p* = 0.25).

### 3.3. Determination of Aphid Species by DNA Barcoding

All collected aphids belonged to the same morphotype also observed during the preliminary investigations on the oak trees. Since DNA barcoding is an efficient and accurate method for the identification of aphids in relation to their morphology, the aphids sampled were used separately to extract DNA and amplify a portion of the COI gene. DNA sequencing resulted in a 658 bp-long COI fragment in all the samples sequenced (*n* = 27, at least one sequence per gall in which aphids were recovered, see Table S1), with sequence variations due to single substitutions only, whereas insertions or deletions were not detected in the analysis (Table S2). Indeed, COI sequence variation within the sampled aphids was very low (sequence identity ranging from 99.7 to 100%), and only two polymorphic sites were observed, defining three haplotypes overall (Table S3). The identification engine available in BOLD identified the COI sequences as belonging to the *Panaphis juglandis* species.

### 3.4. Inner Architecture: Excavation Volume and 2D Analysis

**Figure 6:**
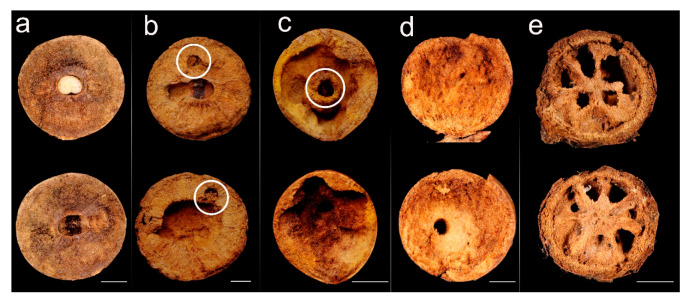
Inner architecture of galls colonized by *C. scutellaris* and *C. truncata*. The effect of different colony composition and the presence of aphids are shown in the two halves of the gall: (**a**) gall still occupied by the cynipid; (**b**) Qa (queen and aphids), showing evidence (circled) of the chamber where the aphids were found (1); (**c**) Qwba (queen, workers, brood and aphids), showing evidence of an aphid chamber (2); (**d**) W, the gall was completely excavated by the ants and presented a main central chamber only; (**e**) gall colonized by a single *C. truncata* queen. Scale bars: 5 mm.

The typical inner architecture of a non-colonized gall presented a characteristic oval-shaped chamber at its center, where the cynipid larva had been located (Figure 6a). Galls occupied by *C. scutellaris* showed different inner architectures, depending on colony composition. 

When only a founding queen with aphids inhabited the gall (Qa), the chamber produced by the cynipid larva was still visible, but a larger area was excavated around it, forming a mid-sized chamber. In addition, a much smaller chamber filled with aphids was also present (Figure 6b). On the other hand, when the gall was occupied by both a queen, workers, brood and aphids (Qwba), an even larger main chamber was found in addition to the small chamber hosting the aphids (Figure 6c). Finally, when the gall was occupied by workers only (W), the amount of excavated material was even larger so that the whole gall served as a single chamber (Figure 6d). 

A peculiar structure was recorded in galls occupied by single queens of *C. truncata*: a radially distributed structure delimiting chambers in the two halves of the gall (Figure 6e). 

**Figure 7:**
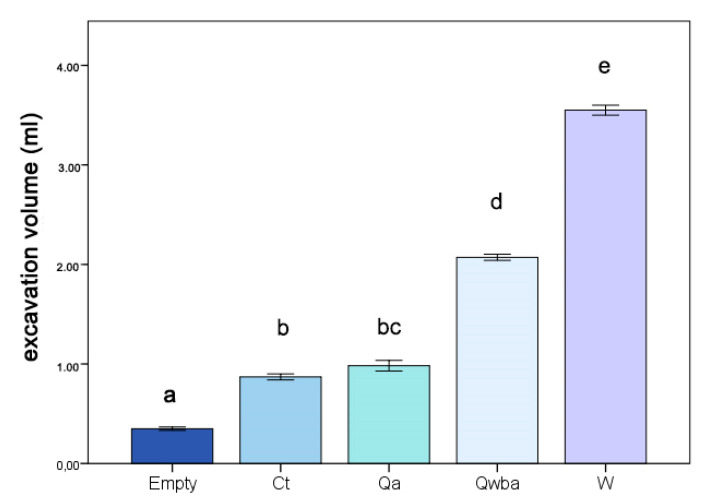
Total excavation volume (mL) in colonized galls according to colony composition: a: empty galls; b: galls with a queen of *C. truncata*; c: galls with a queen of *C. scutellaris* and aphids (Qa); d: galls with queen, workers, brood of *C. scutellaris* and aphids (Qwba); e: galls with workers of *C. scutellaris* only. The SE interval is shown for each bar. The bars with the same letter are not statistically different (one-way ANOVA, see text for further details).

The analysis of the excavation volume in galls colonized by ants showed significant differences between the categories of colony composition (one-way ANOVA: F_4,17_ = 758.13; *p* < 0.001). Tukey’s tests showed four different groups, with the largest volumes being associated with galls with workers only (W), and a queen, workers, brood and aphids (Qwba) of *C. scutellaris*. Moreover, there was no difference between *C. scutellaris* queen and aphid, and queen of *C. truncata* only (Mean ± SE; empty 0.34 ± 0.03 mL; Qa 0.98 ± 0.05 mL; Qwba 2.07 ± 0.03 mL; W 3.55 ± 0.05 mL) (Figure 7). The smallest volume inside the gall was recorded for empty galls (Figure 7). No differences were detected in excavation volume analysis of the aphid chamber in galls colonized by *C. scutellaris* with a queen only, and with a queen, workers and brood (one-way ANOVA: F_1,9_ = 122.85; *p* = 0.610; Mean ± SE; Qa 0.10 ± 0.006 mL; Qwba 0.14 ± 0.047 mL). 

**Figure 8:**
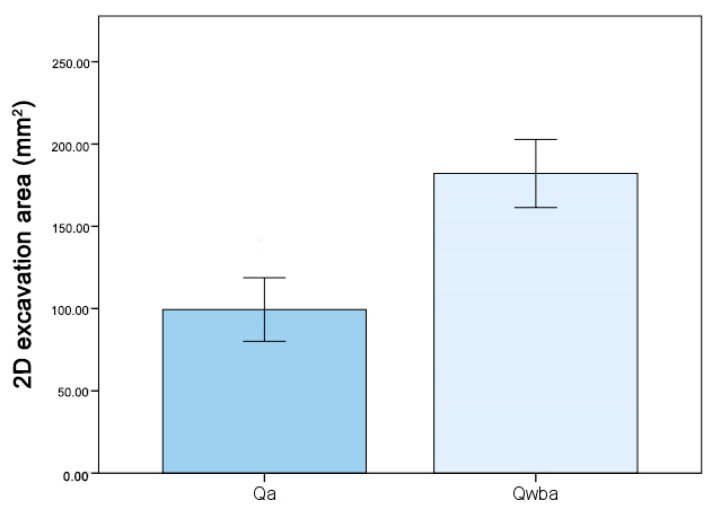
Two-dimensional excavation area (mm^2^) of the ant chamber in experimental galls of *C. scutellaris* containing aphids (*P. juglandis*). Qa = queen only + aphids; Qwba = *C. scutellaris* queen + workers + brood + aphids. SE interval is shown for each bar. The bars with the same letter are not statistically different (one-way ANOVA, Tukey’s post-hoc tests).

Further analyses on nest architecture were focused on galls with aphids only. The analysis of the total 2D excavation area of the ant chamber found significant differences among the three categories of colony composition (one-way ANOVA: F_1,9_ = 6.98, *p* = 0.027). Tukey’s post-hoc tests showed three different groups (Figure 8). The Qwba galls showed the largest excavated area, while empty galls showed the smallest one, and Qa galls were in between (Mean ± SE; Qa 99.36 ± 19.3 mm^2^; Qwba 182.10 ± 20.67 mm^2^). 

**Figure 9:**
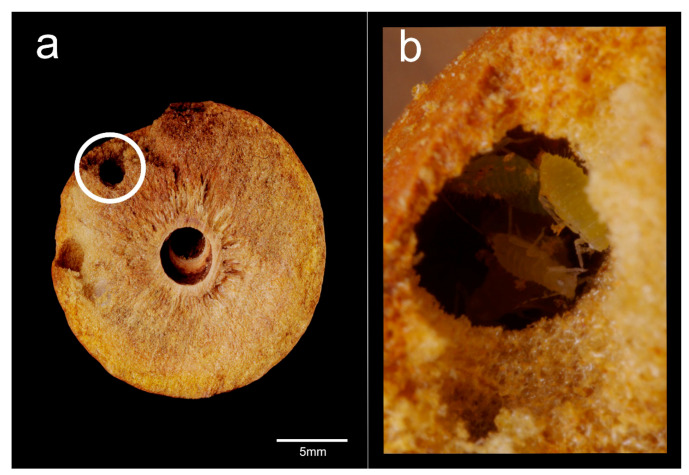
(**a**) Section of a gall colonized by a single queen of *C. scutellaris*; the circle highlights the aphid chamber; (**b**) aphids present inside the chamber excavated by the queen.

**Figure 10:**
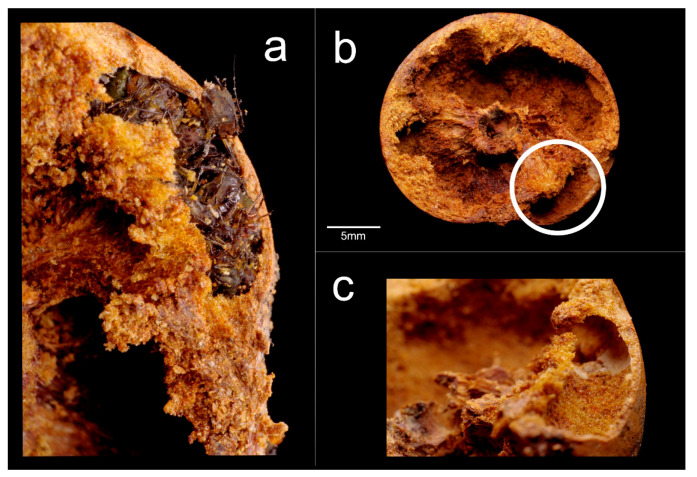
(**a**) Aphids inside the chamber in a Qwba gall nest of *C. scutellaris*—all aphids were still alive at the moment of checking; (**b**) section of the gall showing the chamber’s elongated shape, highlighted by the circle; (**c**) detail of the entrance hole connecting the aphid chamber to the rest of the excavated area occupied by the ants.

The analysis of the galls showed that the aphids were only located in specific small chambers (Figure 9a,b and Figure 10a) connected to the main excavated area (Figure 10b,c). As seen before, the statistical analysis of the aphid chamber size showed non-significant differences among the different ant colony compositions (Mean ± SE; Qa 9.8 ± 3.2 mm^2^; Qwba 10.5 ± 2.8 mm^2^, one-way ANOVA: F_1,10_ = 0.02, *p* = 0.89). 

## 4. Discussion

We recently showed that ants and plants may engage in a sort of by-product mutualism based on oak gall colonization by ants, *C. scutellaris* being a very frequent species in this relationship [4]. In particular, oak galls, which are induced by plant parasites, have an obvious detrimental effect on plant fitness. However, once they have been colonized by ants, they may become a resource for the plant, granting important benefits due to the indirect defense which ants provide against predators and pathogens [4,43]. Given their position and persistence on the plant, once abandoned by the original occupant, these kinds of galls may be considered functionally similar to “*domatia*” of myrmecophytes facilitating the foundation of ant colonies on plants [4]. 

In the present research, we further assessed that *C. scutellaris* is a common inhabitant of abandoned oak galls (in this case induced by the cynipid wasp *A. kollari*) and may process the inner portion in a specific way depending on colony composition. Both the survey and field experiment showed that *C. scutellaris* mainly colonizes galls located in a higher position on the plant, while the other “gall ants” present at the same site (*T. italicus* and *C. truncata*) were confined to a lower height from the ground. The segregation observed on the trees may not be the result of a specific preference by the ants but just the result of the competitive ability of the acrobat ant *C. scutellaris*. This ant is ranked among the strongest competitor in Mediterranean regions and is able to strongly affect the presence of other ant species in the area [44,45,46,47,48]. The presence of complete colonies of this ant species within galls experimentally placed for one year may have been the product of new colony foundations or colony relocations (including from other galls, see [4]). 

Our results are consistent with previous data on colonization by *C. scutellaris* of galls induced by another cynipid, *Andricus quercustozae* (Bosc, 1792) [4]. However, the interesting insect–plant relationship that galls mediate may be more complex than previously documented, as our study reports the involvement of aphids for the first time. The discovery of aphid presence within galls colonized by *C. scutellaris* in special “aphid chambers” can be interpreted through two alternative explanations. One is that *C. scutellaris* actively transport the live aphids from the outside and store them within their gall nests, excavating a dedicated chamber for this purpose. The other alternative is that aphids spontaneously move into galls inhabited by this ant species. However, we consider this second explanation very unlikely: aphids have no ability to excavate galls (i.e., cannot have built “aphid chambers” themselves) and their locomotion ability is normally remarkably modest (so that dispersal is achieved by flying forms), while active relocations of mutualist aphids or coccids by ants is a documented phenomenon [49,50]. 

While oak galls may offer a shelter to *P. juglandis* aphids, they cannot be considered as a source of nutrients for sap-feeders, and it is, therefore, unlikely to think that the aphids found in the galls collected in autumn have been there already during their activity season (spring or summer). Since mutualistic aphids may represent an important source of energy for the colony through honeydew production [11], a hypothesis is that ants store them so that they can safely overwinter and constitute a readily usable resource as honeydew producers at the beginning of the new season, at the restart of the plants’ vegetative activity in spring. *Panaphis juglandis* is an aphid species that usually overwinters as eggs laid on walnut trees. After egg hatching in spring, the *P. juglandis* foundress produces large numbers of *alate viviparae* which colonize the upper sides of walnut leaves along the veins [51,52]. *P. juglandis* aphids is reported to engage in mutualistic relationships with several species of ants that frequently antennate them to encourage honeydew production [53,54,55,56,57]. Moreover, ants even seem to be involved in the protection of this species from damage indirectly caused by other aphids such as *Chromaphis juglandicola* (Kaltenbach, 1843). Indeed, differently from *P. juglandis*, *C. juglandicola* lives in scattered colonies on the bottom part of the leaf blade [58] and causes a constant drip of honeydew and debris to the upper leaf surface colonized by *P. juglandis* inhibiting its reproduction [59]. Interestingly, ants of the species *Lasius niger* (Linnaeus, 1758) and *Myrmica ruginodis* (Nylander, 1846) control the population size of *C. juglandicola*, indirectly protecting their partner [23]. Our current investigation suggests that the entire life cycle of *P. juglandis* can develop on oaks, and not on walnut trees only (considering they were also found elsewhere on the oaks’ branches), and also provides further proof of a well-developed relationship between these aphids and ants, which may offer the former an alternative strategy to overwinter. 

At the same time, we cannot exclude the possibility that at least some of the stored aphids are consumed as food by ant colonies at different developing stages, and that keeping them alive before consumption is aimed at preventing the deterioration of such a resource. Different studies have shown that nutritional needs and the growth in aphid population lead different ant species, such as *Oecophylla longinoda* (Latreille, 1802), *L. flavus* and *L. niger* (Linnaeus, 1758), to increase predation on the aphid colonies attended [60,61]. However, in temperate areas such as the one where our study was conducted, ants such as *C. scutellaris* tend to cease most activities during winter, and *C. scutellaris* foundresses produce their first workers in spring. As a result, aphids would probably be consumed only at a time when they could also be employed as honeydew-producers.

The presence of multiple aphids and of an “aphid chamber,” even in galls occupied only by queens of *C. scutellaris*, suggests that queens must perform multiple sorties during the first stages of gall colonization to transport the aphids. Whether aphids are then to serve as food for the queen itself or for its brood (as preys), or later on for the queen and the workers’ incipient colony (as honeydew producers) appears to be a significant break-away from the current assumption of complete claustrality during colony foundation of *C. scutellaris*. Claustral foundation is thought to be the prevalent strategy in free-living European ant species performing long-range dispersal for colony foundation (i.e., not colony budding) [38], and it requires the foundress queen to never leave its nest chamber and feed its first brood by solely employing its own physiological reserves of nutrients. However, the assumption that ant queens possess bodies evidently adapted for fat storage to perform a strictly claustral colony foundation has not been intensively investigated and remains a mere hypothesis for most species. 

## 5. Conclusions

Ant colonization of galls is a widespread phenomenon that has been extensively overlooked, but it appears to be particularly interesting when considering the recognized ecological and evolutionary importance of the complex and diverse ant-plants interactions. The discovery of live aphids in special chambers within ant-colonized galls further increases the variety of documented mutualistic interactions that ants can establish with their insect partners, confirming their role as keystone organisms [6,62].

Further investigation is required to establish the ultimate fate of these aphids. We support the hypothesis that at least part of them survives the winter, and this could open new interesting possibilities for the life cycle of *P. juglandis*, possibly influencing its population dynamics by allowing an alternative overwintering strategy. Moreover, our results also encourage targeted investigation on foundress queen behavior in those ant species that have been traditionally assumed to be strictly claustral, but which may instead perform interesting activities outside their nest during such a delicate phase of their life cycle. Finally, it is also worth noting that previous intensive surveys on the same area discovered no aphids within galls induced by a different cynipid (*A. quercustozae*) and occupied by the same ant species. New efforts should try to quantify the consistence of this newly discovered ant-aphid relationship, and establish whether and how characteristics of different galls or cynipids may play a meaningful role.

## Data Availability

Data is contained within the article or supplementary material.

## Supplementary Materials

The following are available online at https://www.mdpi.com/2075-4450/12/2/108/s1, Table S1: *P. juglandis* COI sequences, Table S2: *P. juglandis* COI sequence alignment performed with *CLUSTAL* and edited with *Mview*, Table S3: Summary of the polymorphic sites in the *P. juglandis* COI sequences, Table S4: data set survey and field experiment.
