# Supplementary material for: First Report on the Acrobat Ant Crematogaster scutellaris Storing Live Aphids in Its Oak-Gall Nests
Source: Insects. 2021 Jan 27;12(2):108. doi: 10.3390/insects12020108 (PMC7912212; doi:10.3390/insects12020108)
Supplement: Supplementary file 1 [file insects-12-00108-s001.zip › Table S1.pdf]

Supplementary file 1. *P. juglandis* COI sequences

>PANJUG.G1.sample1_COI
AACTTTATATTTCTTATTTGGAATTTGAGCAGGAATAATCGGATCTTCTTTAAGAATTTTAATTCGATTAGAA
TTAAGCCAAATTAATTCAATTATTAATAATAACCAATTATATAATGTAATTGTAACAATTCATGCTTTTATTA
TAATTTTTTTTATAACTATACCTATTGTAATTGGAGGGTTTGGAAATTGATTAATTCCATTAATAATAGGATG
TCCAGATATATCATTTCCACGATTAAATAATATTAGATTTTGACTTTTACCTCCATCTTTAATAATAATAATT
TCTAGATTTTTAATTAATAATGGTACAGGAACAGGTTGAACAATTTATCCACCTCTCTCAAATAACATTGCAC
ATAATAATATTTCAGTTGATTTAACAATTTTCTCATTACACTTAGCAGGAATCTCATCAATTTTAGGAGCTAT
TAATTTTATTTGTACAATTCTTAATATAATACCAAATAATTTAAAATTAAATCAAATTCCATTATTTCCCTGA
TCAATTTTAATTACTGCTATTTTACTAATTATTTCACTACCAGTTTTAGCTGGTGCCATTACAATACTATTAA
CTGACCGAAACCTAAATACATCTTTTTTTGATCCATCAGGAGGAGGTGATCCAATTTTATATCAACATTTATT
C

>PANJUG.G1.sample2_COI
AACTTTATATTTCTTATTTGGAATTTGAGCAGGAATAATCGGATCTTCTTTAAGAATTTTAATTCGATTAGAA
TTAAGCCAAATTAATTCAATTATTAATAATAACCAATTATATAATGTAATTGTAACAATTCATGCTTTTATTA
TAATTTTTTTTATAACTATACCTATTGTAATTGGAGGGTTTGGAAATTGATTAATTCCATTAATAATAGGATG
TCCAGATATATCATTTCCACGATTAAATAATATTAGATTTTGACTTTTACCTCCATCTTTAATAATAATAATT
TCTAGATTTTTAATTAATAATGGTACAGGAACAGGTTGAACAATTTATCCACCTCTCTCAAATAACATTGCAC
ATAATAATATTTCAGTTGATTTAACAATTTTCTCATTACACTTAGCAGGAATCTCATCAATTTTAGGAGCTAT
TAATTTTATTTGTACAATTCTTAATATAATACCAAATAATTTAAAATTAAATCAAATTCCATTATTTCCCTGA
TCAATTTTAATTACTGCTATTTTACTAATTATTTCACTACCAGTTTTAGCTGGTGCCATTACAATACTATTAA
CTGACCGAAACCTAAATACATCTTTTTTTGATCCATCAGGAGGAGGTGATCCAATTTTATATCAACATTTATT
C

>PANJUG.G2.sample1_COI
AACATTATATTTCTTATTTGGAATTTGAGCAGGAATAATCGGATCTTCTTTAAGAATTTTAATTCGATTAGAA
TTAAGCCAAATTAATTCAATTATTAATAATAACCAATTATATAATGTAATTGTAACAATTCATGCTTTTATTA
TAATTTTTTTTATAACTATACCTATTGTAATTGGAGGGTTTGGAAATTGATTAATTCCATTAATAATAGGATG
TCCAGATATATCATTTCCACGATTAAATAATATTAGATTTTGACTTTTACCTCCATCTTTAATAATAATAATT
TCTAGATTTTTAATTAATAATGGTACAGGAACAGGTTGAACAATTTATCCACCTCTCTCAAATAACATTGCAC
ATAATAATATTTCAGTTGATTTAACAATTTTCTCATTACACTTAGCAGGAATCTCATCAATTTTAGGAGCTAT
TAATTTTATTTGTACAATTCTTAATATAATACCAAATAATTTAAAATTAAATCAAATTCCATTATTTCCCTGA
TCAATTTTAATTACTGCTATTTTACTAATTATTTCACTACCAGTTTTAGCTGGTGCCATTACAATACTATTAA
CTGACCGAAACCTAAATACATCTTTTTTTGATCCATCAGGAGGAGGTGATCCAATTTTATATCAACATTTATT
T

>PANJUG.G2.sample2_COI
AACATTATATTTCTTATTTGGAATTTGAGCAGGAATAATCGGATCTTCTTTAAGAATTTTAATTCGATTAGAA
TTAAGCCAAATTAATTCAATTATTAATAATAACCAATTATATAATGTAATTGTAACAATTCATGCTTTTATTA
TAATTTTTTTTATAACTATACCTATTGTAATTGGAGGGTTTGGAAATTGATTAATTCCATTAATAATAGGATG
TCCAGATATATCATTTCCACGATTAAATAATATTAGATTTTGACTTTTACCTCCATCTTTAATAATAATAATT
TCTAGATTTTTAATTAATAATGGTACAGGAACAGGTTGAACAATTTATCCACCTCTCTCAAATAACATTGCAC
ATAATAATATTTCAGTTGATTTAACAATTTTCTCATTACACTTAGCAGGAATCTCATCAATTTTAGGAGCTAT
TAATTTTATTTGTACAATTCTTAATATAATACCAAATAATTTAAAATTAAATCAAATTCCATTATTTCCCTGA
TCAATTTTAATTACTGCTATTTTACTAATTATTTCACTACCAGTTTTAGCTGGTGCCATTACAATACTATTAA
CTGACCGAAACCTAAATACATCTTTTTTTGATCCATCAGGAGGAGGTGATCCAATTTTATATCAACATTTATT
T

>PANJUG.G2.sample3_COI
AACATTATATTTCTTATTTGGAATTTGAGCAGGAATAATCGGATCTTCTTTAAGAATTTTAATTCGATTAGAA
TTAAGCCAAATTAATTCAATTATTAATAATAACCAATTATATAATGTAATTGTAACAATTCATGCTTTTATTA
TAATTTTTTTTATAACTATACCTATTGTAATTGGAGGGTTTGGAAATTGATTAATTCCATTAATAATAGGATG
TCCAGATATATCATTTCCACGATTAAATAATATTAGATTTTGACTTTTACCTCCATCTTTAATAATAATAATT
TCTAGATTTTTAATTAATAATGGTACAGGAACAGGTTGAACAATTTATCCACCTCTCTCAAATAACATTGCAC
ATAATAATATTTCAGTTGATTTAACAATTTTCTCATTACACTTAGCAGGAATCTCATCAATTTTAGGAGCTAT
TAATTTTATTTGTACAATTCTTAATATAATACCAAATAATTTAAAATTAAATCAAATTCCATTATTTCCCTGA

TCAATTTTAATTACTGCTATTTTACTAATTATTTCACTACCAGTTTTAGCTGGTGCCATTACAATACTATTAA
CTGACCGAAACCTAAATACATCTTTTTTTGATCCATCAGGAGGAGGTGATCCAATTTTATATCAACATTTATT
T

>PANJUG.G3.sample1_COI
AACTTTATATTTCTTATTTGGAATTTGAGCAGGAATAATCGGATCTTCTTTAAGAATTTTAATTCGATTAGAA
TTAAGCCAAATTAATTCAATTATTAATAATAACCAATTATATAATGTAATTGTAACAATTCATGCTTTTATTA
TAATTTTTTTTATAACTATACCTATTGTAATTGGAGGGTTTGGAAATTGATTAATTCCATTAATAATAGGATG
TCCAGATATATCATTTCCACGATTAAATAATATTAGATTTTGACTTTTACCTCCATCTTTAATAATAATAATT
TCTAGATTTTTAATTAATAATGGTACAGGAACAGGTTGAACAATTTATCCACCTCTCTCAAATAACATTGCAC
ATAATAATATTTCAGTTGATTTAACAATTTTCTCATTACACTTAGCAGGAATCTCATCAATTTTAGGAGCTAT
TAATTTTATTTGTACAATTCTTAATATAATACCAAATAATTTAAAATTAAATCAAATTCCATTATTTCCCTGA
TCAATTTTAATTACTGCTATTTTACTAATTATTTCACTACCAGTTTTAGCTGGTGCCATTACAATACTATTAA
CTGACCGAAACCTAAATACATCTTTTTTTGATCCATCAGGAGGAGGTGATCCAATTTTATATCAACATTTATT
C

>PANJUG.G3.sample2_COI
AACTTTATATTTCTTATTTGGAATTTGAGCAGGAATAATCGGATCTTCTTTAAGAATTTTAATTCGATTAGAA
TTAAGCCAAATTAATTCAATTATTAATAATAACCAATTATATAATGTAATTGTAACAATTCATGCTTTTATTA
TAATTTTTTTTATAACTATACCTATTGTAATTGGAGGGTTTGGAAATTGATTAATTCCATTAATAATAGGATG
TCCAGATATATCATTTCCACGATTAAATAATATTAGATTTTGACTTTTACCTCCATCTTTAATAATAATAATT
TCTAGATTTTTAATTAATAATGGTACAGGAACAGGTTGAACAATTTATCCACCTCTCTCAAATAACATTGCAC
ATAATAATATTTCAGTTGATTTAACAATTTTCTCATTACACTTAGCAGGAATCTCATCAATTTTAGGAGCTAT
TAATTTTATTTGTACAATTCTTAATATAATACCAAATAATTTAAAATTAAATCAAATTCCATTATTTCCCTGA
TCAATTTTAATTACTGCTATTTTACTAATTATTTCACTACCAGTTTTAGCTGGTGCCATTACAATACTATTAA
CTGACCGAAACCTAAATACATCTTTTTTTGATCCATCAGGAGGAGGTGATCCAATTTTATATCAACATTTATT
C

>PANJUG.G3.sample3_COI
AACTTTATATTTCTTATTTGGAATTTGAGCAGGAATAATCGGATCTTCTTTAAGAATTTTAATTCGATTAGAA
TTAAGCCAAATTAATTCAATTATTAATAATAACCAATTATATAATGTAATTGTAACAATTCATGCTTTTATTA
TAATTTTTTTTATAACTATACCTATTGTAATTGGAGGGTTTGGAAATTGATTAATTCCATTAATAATAGGATG
TCCAGATATATCATTTCCACGATTAAATAATATTAGATTTTGACTTTTACCTCCATCTTTAATAATAATAATT
TCTAGATTTTTAATTAATAATGGTACAGGAACAGGTTGAACAATTTATCCACCTCTCTCAAATAACATTGCAC
ATAATAATATTTCAGTTGATTTAACAATTTTCTCATTACACTTAGCAGGAATCTCATCAATTTTAGGAGCTAT
TAATTTTATTTGTACAATTCTTAATATAATACCAAATAATTTAAAATTAAATCAAATTCCATTATTTCCCTGA
TCAATTTTAATTACTGCTATTTTACTAATTATTTCACTACCAGTTTTAGCTGGTGCCATTACAATACTATTAA
CTGACCGAAACCTAAATACATCTTTTTTTGATCCATCAGGAGGAGGTGATCCAATTTTATATCAACATTTATT
C

>PANJUG.G3.sample4_COI
AACATTATATTTCTTATTTGGAATTTGAGCAGGAATAATCGGATCTTCTTTAAGAATTTTAATTCGATTAGAA
TTAAGCCAAATTAATTCAATTATTAATAATAACCAATTATATAATGTAATTGTAACAATTCATGCTTTTATTA
TAATTTTTTTTATAACTATACCTATTGTAATTGGAGGGTTTGGAAATTGATTAATTCCATTAATAATAGGATG
TCCAGATATATCATTTCCACGATTAAATAATATTAGATTTTGACTTTTACCTCCATCTTTAATAATAATAATT
TCTAGATTTTTAATTAATAATGGTACAGGAACAGGTTGAACAATTTATCCACCTCTCTCAAATAACATTGCAC
ATAATAATATTTCAGTTGATTTAACAATTTTCTCATTACACTTAGCAGGAATCTCATCAATTTTAGGAGCTAT
TAATTTTATTTGTACAATTCTTAATATAATACCAAATAATTTAAAATTAAATCAAATTCCATTATTTCCCTGA
TCAATTTTAATTACTGCTATTTTACTAATTATTTCACTACCAGTTTTAGCTGGTGCCATTACAATACTATTAA
CTGACCGAAACCTAAATACATCTTTTTTTGATCCATCAGGAGGAGGTGATCCAATTTTATATCAACATTTATT
T

>PANJUG.G3.sample5_COI
AACATTATATTTCTTATTTGGAATTTGAGCAGGAATAATCGGATCTTCTTTAAGAATTTTAATTCGATTAGAA
TTAAGCCAAATTAATTCAATTATTAATAATAACCAATTATATAATGTAATTGTAACAATTCATGCTTTTATTA
TAATTTTTTTTATAACTATACCTATTGTAATTGGAGGGTTTGGAAATTGATTAATTCCATTAATAATAGGATG
TCCAGATATATCATTTCCACGATTAAATAATATTAGATTTTGACTTTTACCTCCATCTTTAATAATAATAATT
TCTAGATTTTTAATTAATAATGGTACAGGAACAGGTTGAACAATTTATCCACCTCTCTCAAATAACATTGCAC
ATAATAATATTTCAGTTGATTTAACAATTTTCTCATTACACTTAGCAGGAATCTCATCAATTTTAGGAGCTAT
TAATTTTATTTGTACAATTCTTAATATAATACCAAATAATTTAAAATTAAATCAAATTCCATTATTTCCCTGA

TCAATTTTAATTACTGCTATTTTACTAATTATTTCACTACCAGTTTTAGCTGGTGCCATTACAATACTATTAA
CTGACCGAAACCTAAATACATCTTTTTTTGATCCATCAGGAGGAGGTGATCCAATTTTATATCAACATTTATT
T

>PANJUG.G3.sample6_COI
AACTTTATATTTCTTATTTGGAATTTGAGCAGGAATAATCGGATCTTCTTTAAGAATTTTAATTCGATTAGAA
TTAAGCCAAATTAATTCAATTATTAATAATAACCAATTATATAATGTAATTGTAACAATTCATGCTTTTATTA
TAATTTTTTTTATAACTATACCTATTGTAATTGGAGGGTTTGGAAATTGATTAATTCCATTAATAATAGGATG
TCCAGATATATCATTTCCACGATTAAATAATATTAGATTTTGACTTTTACCTCCATCTTTAATAATAATAATT
TCTAGATTTTTAATTAATAATGGTACAGGAACAGGTTGAACAATTTATCCACCTCTCTCAAATAACATTGCAC
ATAATAATATTTCAGTTGATTTAACAATTTTCTCATTACACTTAGCAGGAATCTCATCAATTTTAGGAGCTAT
TAATTTTATTTGTACAATTCTTAATATAATACCAAATAATTTAAAATTAAATCAAATTCCATTATTTCCCTGA
TCAATTTTAATTACTGCTATTTTACTAATTATTTCACTACCAGTTTTAGCTGGTGCCATTACAATACTATTAA
CTGACCGAAACCTAAATACATCTTTTTTTGATCCATCAGGAGGAGGTGATCCAATTTTATATCAACATTTATT
T

>PANJUG.G3.sample7_COI
AACTTTATATTTCTTATTTGGAATTTGAGCAGGAATAATCGGATCTTCTTTAAGAATTTTAATTCGATTAGAA
TTAAGCCAAATTAATTCAATTATTAATAATAACCAATTATATAATGTAATTGTAACAATTCATGCTTTTATTA
TAATTTTTTTTATAACTATACCTATTGTAATTGGAGGGTTTGGAAATTGATTAATTCCATTAATAATAGGATG
TCCAGATATATCATTTCCACGATTAAATAATATTAGATTTTGACTTTTACCTCCATCTTTAATAATAATAATT
TCTAGATTTTTAATTAATAATGGTACAGGAACAGGTTGAACAATTTATCCACCTCTCTCAAATAACATTGCAC
ATAATAATATTTCAGTTGATTTAACAATTTTCTCATTACACTTAGCAGGAATCTCATCAATTTTAGGAGCTAT
TAATTTTATTTGTACAATTCTTAATATAATACCAAATAATTTAAAATTAAATCAAATTCCATTATTTCCCTGA
TCAATTTTAATTACTGCTATTTTACTAATTATTTCACTACCAGTTTTAGCTGGTGCCATTACAATACTATTAA
CTGACCGAAACCTAAATACATCTTTTTTTGATCCATCAGGAGGAGGTGATCCAATTTTATATCAACATTTATT
C

>PANJUG.G3.sample8_COI
AACTTTATATTTCTTATTTGGAATTTGAGCAGGAATAATCGGATCTTCTTTAAGAATTTTAATTCGATTAGAA
TTAAGCCAAATTAATTCAATTATTAATAATAACCAATTATATAATGTAATTGTAACAATTCATGCTTTTATTA
TAATTTTTTTTATAACTATACCTATTGTAATTGGAGGGTTTGGAAATTGATTAATTCCATTAATAATAGGATG
TCCAGATATATCATTTCCACGATTAAATAATATTAGATTTTGACTTTTACCTCCATCTTTAATAATAATAATT
TCTAGATTTTTAATTAATAATGGTACAGGAACAGGTTGAACAATTTATCCACCTCTCTCAAATAACATTGCAC
ATAATAATATTTCAGTTGATTTAACAATTTTCTCATTACACTTAGCAGGAATCTCATCAATTTTAGGAGCTAT
TAATTTTATTTGTACAATTCTTAATATAATACCAAATAATTTAAAATTAAATCAAATTCCATTATTTCCCTGA
TCAATTTTAATTACTGCTATTTTACTAATTATTTCACTACCAGTTTTAGCTGGTGCCATTACAATACTATTAA
CTGACCGAAACCTAAATACATCTTTTTTTGATCCATCAGGAGGAGGTGATCCAATTTTATATCAACATTTATT
C

>PANJUG.G3.sample9_COI
AACTTTATATTTCTTATTTGGAATTTGAGCAGGAATAATCGGATCTTCTTTAAGAATTTTAATTCGATTAGAA
TTAAGCCAAATTAATTCAATTATTAATAATAACCAATTATATAATGTAATTGTAACAATTCATGCTTTTATTA
TAATTTTTTTTATAACTATACCTATTGTAATTGGAGGGTTTGGAAATTGATTAATTCCATTAATAATAGGATG
TCCAGATATATCATTTCCACGATTAAATAATATTAGATTTTGACTTTTACCTCCATCTTTAATAATAATAATT
TCTAGATTTTTAATTAATAATGGTACAGGAACAGGTTGAACAATTTATCCACCTCTCTCAAATAACATTGCAC
ATAATAATATTTCAGTTGATTTAACAATTTTCTCATTACACTTAGCAGGAATCTCATCAATTTTAGGAGCTAT
TAATTTTATTTGTACAATTCTTAATATAATACCAAATAATTTAAAATTAAATCAAATTCCATTATTTCCCTGA
TCAATTTTAATTACTGCTATTTTACTAATTATTTCACTACCAGTTTTAGCTGGTGCCATTACAATACTATTAA
CTGACCGAAACCTAAATACATCTTTTTTTGATCCATCAGGAGGAGGTGATCCAATTTTATATCAACATTTATT
C

>PANJUG.G3.sample10_COI
AACTTTATATTTCTTATTTGGAATTTGAGCAGGAATAATCGGATCTTCTTTAAGAATTTTAATTCGATTAGAA
TTAAGCCAAATTAATTCAATTATTAATAATAACCAATTATATAATGTAATTGTAACAATTCATGCTTTTATTA
TAATTTTTTTTATAACTATACCTATTGTAATTGGAGGGTTTGGAAATTGATTAATTCCATTAATAATAGGATG
TCCAGATATATCATTTCCACGATTAAATAATATTAGATTTTGACTTTTACCTCCATCTTTAATAATAATAATT
TCTAGATTTTTAATTAATAATGGTACAGGAACAGGTTGAACAATTTATCCACCTCTCTCAAATAACATTGCAC
ATAATAATATTTCAGTTGATTTAACAATTTTCTCATTACACTTAGCAGGAATCTCATCAATTTTAGGAGCTAT
TAATTTTATTTGTACAATTCTTAATATAATACCAAATAATTTAAAATTAAATCAAATTCCATTATTTCCCTGA

TCAATTTTAATTACTGCTATTTTACTAATTATTTCACTACCAGTTTTAGCTGGTGCCATTACAATACTATTAA
CTGACCGAAACCTAAATACATCTTTTTTTGATCCATCAGGAGGAGGTGATCCAATTTTATATCAACATTTATT
C

>PANJUG.G3.sample11_COI
AACATTATATTTCTTATTTGGAATTTGAGCAGGAATAATCGGATCTTCTTTAAGAATTTTAATTCGATTAGAA
TTAAGCCAAATTAATTCAATTATTAATAATAACCAATTATATAATGTAATTGTAACAATTCATGCTTTTATTA
TAATTTTTTTTATAACTATACCTATTGTAATTGGAGGGTTTGGAAATTGATTAATTCCATTAATAATAGGATG
TCCAGATATATCATTTCCACGATTAAATAATATTAGATTTTGACTTTTACCTCCATCTTTAATAATAATAATT
TCTAGATTTTTAATTAATAATGGTACAGGAACAGGTTGAACAATTTATCCACCTCTCTCAAATAACATTGCAC
ATAATAATATTTCAGTTGATTTAACAATTTTCTCATTACACTTAGCAGGAATCTCATCAATTTTAGGAGCTAT
TAATTTTATTTGTACAATTCTTAATATAATACCAAATAATTTAAAATTAAATCAAATTCCATTATTTCCCTGA
TCAATTTTAATTACTGCTATTTTACTAATTATTTCACTACCAGTTTTAGCTGGTGCCATTACAATACTATTAA
CTGACCGAAACCTAAATACATCTTTTTTTGATCCATCAGGAGGAGGTGATCCAATTTTATATCAACATTTATT
T

>PANJUG.G3.sample12_COI
AACATTATATTTCTTATTTGGAATTTGAGCAGGAATAATCGGATCTTCTTTAAGAATTTTAATTCGATTAGAA
TTAAGCCAAATTAATTCAATTATTAATAATAACCAATTATATAATGTAATTGTAACAATTCATGCTTTTATTA
TAATTTTTTTTATAACTATACCTATTGTAATTGGAGGGTTTGGAAATTGATTAATTCCATTAATAATAGGATG
TCCAGATATATCATTTCCACGATTAAATAATATTAGATTTTGACTTTTACCTCCATCTTTAATAATAATAATT
TCTAGATTTTTAATTAATAATGGTACAGGAACAGGTTGAACAATTTATCCACCTCTCTCAAATAACATTGCAC
ATAATAATATTTCAGTTGATTTAACAATTTTCTCATTACACTTAGCAGGAATCTCATCAATTTTAGGAGCTAT
TAATTTTATTTGTACAATTCTTAATATAATACCAAATAATTTAAAATTAAATCAAATTCCATTATTTCCCTGA
TCAATTTTAATTACTGCTATTTTACTAATTATTTCACTACCAGTTTTAGCTGGTGCCATTACAATACTATTAA
CTGACCGAAACCTAAATACATCTTTTTTTGATCCATCAGGAGGAGGTGATCCAATTTTATATCAACATTTATT
T

>PANJUG.G3.sample13_COI
AACTTTATATTTCTTATTTGGAATTTGAGCAGGAATAATCGGATCTTCTTTAAGAATTTTAATTCGATTAGAA
TTAAGCCAAATTAATTCAATTATTAATAATAACCAATTATATAATGTAATTGTAACAATTCATGCTTTTATTA
TAATTTTTTTTATAACTATACCTATTGTAATTGGAGGGTTTGGAAATTGATTAATTCCATTAATAATAGGATG
TCCAGATATATCATTTCCACGATTAAATAATATTAGATTTTGACTTTTACCTCCATCTTTAATAATAATAATT
TCTAGATTTTTAATTAATAATGGTACAGGAACAGGTTGAACAATTTATCCACCTCTCTCAAATAACATTGCAC
ATAATAATATTTCAGTTGATTTAACAATTTTCTCATTACACTTAGCAGGAATCTCATCAATTTTAGGAGCTAT
TAATTTTATTTGTACAATTCTTAATATAATACCAAATAATTTAAAATTAAATCAAATTCCATTATTTCCCTGA
TCAATTTTAATTACTGCTATTTTACTAATTATTTCACTACCAGTTTTAGCTGGTGCCATTACAATACTATTAA
CTGACCGAAACCTAAATACATCTTTTTTTGATCCATCAGGAGGAGGTGATCCAATTTTATATCAACATTTATT
T

>PANJUG.G3.sample14_COI
AACTTTATATTTCTTATTTGGAATTTGAGCAGGAATAATCGGATCTTCTTTAAGAATTTTAATTCGATTAGAA
TTAAGCCAAATTAATTCAATTATTAATAATAACCAATTATATAATGTAATTGTAACAATTCATGCTTTTATTA
TAATTTTTTTTATAACTATACCTATTGTAATTGGAGGGTTTGGAAATTGATTAATTCCATTAATAATAGGATG
TCCAGATATATCATTTCCACGATTAAATAATATTAGATTTTGACTTTTACCTCCATCTTTAATAATAATAATT
TCTAGATTTTTAATTAATAATGGTACAGGAACAGGTTGAACAATTTATCCACCTCTCTCAAATAACATTGCAC
ATAATAATATTTCAGTTGATTTAACAATTTTCTCATTACACTTAGCAGGAATCTCATCAATTTTAGGAGCTAT
TAATTTTATTTGTACAATTCTTAATATAATACCAAATAATTTAAAATTAAATCAAATTCCATTATTTCCCTGA
TCAATTTTAATTACTGCTATTTTACTAATTATTTCACTACCAGTTTTAGCTGGTGCCATTACAATACTATTAA
CTGACCGAAACCTAAATACATCTTTTTTTGATCCATCAGGAGGAGGTGATCCAATTTTATATCAACATTTATT
C

>PANJUG.G3.sample15_COI
AACTTTATATTTCTTATTTGGAATTTGAGCAGGAATAATCGGATCTTCTTTAAGAATTTTAATTCGATTAGAA
TTAAGCCAAATTAATTCAATTATTAATAATAACCAATTATATAATGTAATTGTAACAATTCATGCTTTTATTA
TAATTTTTTTTATAACTATACCTATTGTAATTGGAGGGTTTGGAAATTGATTAATTCCATTAATAATAGGATG
TCCAGATATATCATTTCCACGATTAAATAATATTAGATTTTGACTTTTACCTCCATCTTTAATAATAATAATT
TCTAGATTTTTAATTAATAATGGTACAGGAACAGGTTGAACAATTTATCCACCTCTCTCAAATAACATTGCAC
ATAATAATATTTCAGTTGATTTAACAATTTTCTCATTACACTTAGCAGGAATCTCATCAATTTTAGGAGCTAT
TAATTTTATTTGTACAATTCTTAATATAATACCAAATAATTTAAAATTAAATCAAATTCCATTATTTCCCTGA

TCAATTTTAATTACTGCTATTTTACTAATTATTTCACTACCAGTTTTAGCTGGTGCCATTACAATACTATTAA
CTGACCGAAACCTAAATACATCTTTTTTTGATCCATCAGGAGGAGGTGATCCAATTTTATATCAACATTTATT
C

>PANJUG.G3.sample16_COI
AACTTTATATTTCTTATTTGGAATTTGAGCAGGAATAATCGGATCTTCTTTAAGAATTTTAATTCGATTAGAA
TTAAGCCAAATTAATTCAATTATTAATAATAACCAATTATATAATGTAATTGTAACAATTCATGCTTTTATTA
TAATTTTTTTTATAACTATACCTATTGTAATTGGAGGGTTTGGAAATTGATTAATTCCATTAATAATAGGATG
TCCAGATATATCATTTCCACGATTAAATAATATTAGATTTTGACTTTTACCTCCATCTTTAATAATAATAATT
TCTAGATTTTTAATTAATAATGGTACAGGAACAGGTTGAACAATTTATCCACCTCTCTCAAATAACATTGCAC
ATAATAATATTTCAGTTGATTTAACAATTTTCTCATTACACTTAGCAGGAATCTCATCAATTTTAGGAGCTAT
TAATTTTATTTGTACAATTCTTAATATAATACCAAATAATTTAAAATTAAATCAAATTCCATTATTTCCCTGA
TCAATTTTAATTACTGCTATTTTACTAATTATTTCACTACCAGTTTTAGCTGGTGCCATTACAATACTATTAA
CTGACCGAAACCTAAATACATCTTTTTTTGATCCATCAGGAGGAGGTGATCCAATTTTATATCAACATTTATT
C

>PANJUG.G3.sample17_COI
AACTTTATATTTCTTATTTGGAATTTGAGCAGGAATAATCGGATCTTCTTTAAGAATTTTAATTCGATTAGAA
TTAAGCCAAATTAATTCAATTATTAATAATAACCAATTATATAATGTAATTGTAACAATTCATGCTTTTATTA
TAATTTTTTTTATAACTATACCTATTGTAATTGGAGGGTTTGGAAATTGATTAATTCCATTAATAATAGGATG
TCCAGATATATCATTTCCACGATTAAATAATATTAGATTTTGACTTTTACCTCCATCTTTAATAATAATAATT
TCTAGATTTTTAATTAATAATGGTACAGGAACAGGTTGAACAATTTATCCACCTCTCTCAAATAACATTGCAC
ATAATAATATTTCAGTTGATTTAACAATTTTCTCATTACACTTAGCAGGAATCTCATCAATTTTAGGAGCTAT
TAATTTTATTTGTACAATTCTTAATATAATACCAAATAATTTAAAATTAAATCAAATTCCATTATTTCCCTGA
TCAATTTTAATTACTGCTATTTTACTAATTATTTCACTACCAGTTTTAGCTGGTGCCATTACAATACTATTAA
CTGACCGAAACCTAAATACATCTTTTTTTGATCCATCAGGAGGAGGTGATCCAATTTTATATCAACATTTATT
C

>PANJUG.G3.sample18_COI
AACATTATATTTCTTATTTGGAATTTGAGCAGGAATAATCGGATCTTCTTTAAGAATTTTAATTCGATTAGAA
TTAAGCCAAATTAATTCAATTATTAATAATAACCAATTATATAATGTAATTGTAACAATTCATGCTTTTATTA
TAATTTTTTTTATAACTATACCTATTGTAATTGGAGGGTTTGGAAATTGATTAATTCCATTAATAATAGGATG
TCCAGATATATCATTTCCACGATTAAATAATATTAGATTTTGACTTTTACCTCCATCTTTAATAATAATAATT
TCTAGATTTTTAATTAATAATGGTACAGGAACAGGTTGAACAATTTATCCACCTCTCTCAAATAACATTGCAC
ATAATAATATTTCAGTTGATTTAACAATTTTCTCATTACACTTAGCAGGAATCTCATCAATTTTAGGAGCTAT
TAATTTTATTTGTACAATTCTTAATATAATACCAAATAATTTAAAATTAAATCAAATTCCATTATTTCCCTGA
TCAATTTTAATTACTGCTATTTTACTAATTATTTCACTACCAGTTTTAGCTGGTGCCATTACAATACTATTAA
CTGACCGAAACCTAAATACATCTTTTTTTGATCCATCAGGAGGAGGTGATCCAATTTTATATCAACATTTATT
T

>PANJUG.G3.sample19_COI
AACATTATATTTCTTATTTGGAATTTGAGCAGGAATAATCGGATCTTCTTTAAGAATTTTAATTCGATTAGAA
TTAAGCCAAATTAATTCAATTATTAATAATAACCAATTATATAATGTAATTGTAACAATTCATGCTTTTATTA
TAATTTTTTTTATAACTATACCTATTGTAATTGGAGGGTTTGGAAATTGATTAATTCCATTAATAATAGGATG
TCCAGATATATCATTTCCACGATTAAATAATATTAGATTTTGACTTTTACCTCCATCTTTAATAATAATAATT
TCTAGATTTTTAATTAATAATGGTACAGGAACAGGTTGAACAATTTATCCACCTCTCTCAAATAACATTGCAC
ATAATAATATTTCAGTTGATTTAACAATTTTCTCATTACACTTAGCAGGAATCTCATCAATTTTAGGAGCTAT
TAATTTTATTTGTACAATTCTTAATATAATACCAAATAATTTAAAATTAAATCAAATTCCATTATTTCCCTGA
TCAATTTTAATTACTGCTATTTTACTAATTATTTCACTACCAGTTTTAGCTGGTGCCATTACAATACTATTAA
CTGACCGAAACCTAAATACATCTTTTTTTGATCCATCAGGAGGAGGTGATCCAATTTTATATCAACATTTATT
T

>PANJUG.G3.sample20_COI
AACTTTATATTTCTTATTTGGAATTTGAGCAGGAATAATCGGATCTTCTTTAAGAATTTTAATTCGATTAGAA
TTAAGCCAAATTAATTCAATTATTAATAATAACCAATTATATAATGTAATTGTAACAATTCATGCTTTTATTA
TAATTTTTTTTATAACTATACCTATTGTAATTGGAGGGTTTGGAAATTGATTAATTCCATTAATAATAGGATG
TCCAGATATATCATTTCCACGATTAAATAATATTAGATTTTGACTTTTACCTCCATCTTTAATAATAATAATT
TCTAGATTTTTAATTAATAATGGTACAGGAACAGGTTGAACAATTTATCCACCTCTCTCAAATAACATTGCAC
ATAATAATATTTCAGTTGATTTAACAATTTTCTCATTACACTTAGCAGGAATCTCATCAATTTTAGGAGCTAT
TAATTTTATTTGTACAATTCTTAATATAATACCAAATAATTTAAAATTAAATCAAATTCCATTATTTCCCTGA

TCAATTTTAATTACTGCTATTTTACTAATTATTTCACTACCAGTTTTAGCTGGTGCCATTACAATACTATTAA
CTGACCGAAACCTAAATACATCTTTTTTTGATCCATCAGGAGGAGGTGATCCAATTTTATATCAACATTTATT
T

>PANJUG.G3.sample21_COI
AACTTTATATTTCTTATTTGGAATTTGAGCAGGAATAATCGGATCTTCTTTAAGAATTTTAATTCGATTAGAA
TTAAGCCAAATTAATTCAATTATTAATAATAACCAATTATATAATGTAATTGTAACAATTCATGCTTTTATTA
TAATTTTTTTTATAACTATACCTATTGTAATTGGAGGGTTTGGAAATTGATTAATTCCATTAATAATAGGATG
TCCAGATATATCATTTCCACGATTAAATAATATTAGATTTTGACTTTTACCTCCATCTTTAATAATAATAATT
TCTAGATTTTTAATTAATAATGGTACAGGAACAGGTTGAACAATTTATCCACCTCTCTCAAATAACATTGCAC
ATAATAATATTTCAGTTGATTTAACAATTTTCTCATTACACTTAGCAGGAATCTCATCAATTTTAGGAGCTAT
TAATTTTATTTGTACAATTCTTAATATAATACCAAATAATTTAAAATTAAATCAAATTCCATTATTTCCCTGA
TCAATTTTAATTACTGCTATTTTACTAATTATTTCACTACCAGTTTTAGCTGGTGCCATTACAATACTATTAA
CTGACCGAAACCTAAATACATCTTTTTTTGATCCATCAGGAGGAGGTGATCCAATTTTATATCAACATTTATT
C

>PANJUG.G3.sample22_COI
AACTTTATATTTCTTATTTGGAATTTGAGCAGGAATAATCGGATCTTCTTTAAGAATTTTAATTCGATTAGAA
TTAAGCCAAATTAATTCAATTATTAATAATAACCAATTATATAATGTAATTGTAACAATTCATGCTTTTATTA
TAATTTTTTTTATAACTATACCTATTGTAATTGGAGGGTTTGGAAATTGATTAATTCCATTAATAATAGGATG
TCCAGATATATCATTTCCACGATTAAATAATATTAGATTTTGACTTTTACCTCCATCTTTAATAATAATAATT
TCTAGATTTTTAATTAATAATGGTACAGGAACAGGTTGAACAATTTATCCACCTCTCTCAAATAACATTGCAC
ATAATAATATTTCAGTTGATTTAACAATTTTCTCATTACACTTAGCAGGAATCTCATCAATTTTAGGAGCTAT
TAATTTTATTTGTACAATTCTTAATATAATACCAAATAATTTAAAATTAAATCAAATTCCATTATTTCCCTGA
TCAATTTTAATTACTGCTATTTTACTAATTATTTCACTACCAGTTTTAGCTGGTGCCATTACAATACTATTAA
CTGACCGAAACCTAAATACATCTTTTTTTGATCCATCAGGAGGAGGTGATCCAATTTTATATCAACATTTATT
C
