# Supplementary material for: First Report on the Acrobat Ant Crematogaster scutellaris Storing Live Aphids in Its Oak-Gall Nests
Source: Insects. 2021 Jan 27;12(2):108. doi: 10.3390/insects12020108 (PMC7912212; doi:10.3390/insects12020108)
Supplement: Supplementary file 1 [file insects-12-00108-s001.zip › Table S2.pdf]

Supplementary file 2. *P. juglandis* COI sequence alignment performed with *CLUSTAL* and edited with *Mview* (nucleotide coloured by identity).
cov: sequence coverage. *pid*: percentage of identity

```
                                 cov     pid   1         .         .         .         .         :         .         .         . 80
 1 PANJUG.G1.sample1_COI   100.0% 100.0%      AACTTTATATTTCTTATTTGGAATTTGAGCAGGAATAATCGGATCTTCTTTAAGAATTTTAATTCGATTAGAATTAAGCC
 2 PANJUG.G1.sample2_COI   100.0% 100.0%      AACTTTATATTTCTTATTTGGAATTTGAGCAGGAATAATCGGATCTTCTTTAAGAATTTTAATTCGATTAGAATTAAGCC
 3 PANJUG.G2.sample1_COI   100.0%  99.7%      AACATTATATTTCTTATTTGGAATTTGAGCAGGAATAATCGGATCTTCTTTAAGAATTTTAATTCGATTAGAATTAAGCC
 4 PANJUG.G2.sample2_COI   100.0%  99.7%      AACATTATATTTCTTATTTGGAATTTGAGCAGGAATAATCGGATCTTCTTTAAGAATTTTAATTCGATTAGAATTAAGCC
 5 PANJUG.G2.sample3_COI   100.0%  99.7%      AACATTATATTTCTTATTTGGAATTTGAGCAGGAATAATCGGATCTTCTTTAAGAATTTTAATTCGATTAGAATTAAGCC
 6 PANJUG.G3.sample10_COI  100.0% 100.0%      AACTTTATATTTCTTATTTGGAATTTGAGCAGGAATAATCGGATCTTCTTTAAGAATTTTAATTCGATTAGAATTAAGCC
 7 PANJUG.G3.sample11_COI  100.0%  99.7%      AACATTATATTTCTTATTTGGAATTTGAGCAGGAATAATCGGATCTTCTTTAAGAATTTTAATTCGATTAGAATTAAGCC
 8 PANJUG.G3.sample12_COI  100.0%  99.7%      AACATTATATTTCTTATTTGGAATTTGAGCAGGAATAATCGGATCTTCTTTAAGAATTTTAATTCGATTAGAATTAAGCC
 9 PANJUG.G3.sample13_COI  100.0%  99.8%      AACTTTATATTTCTTATTTGGAATTTGAGCAGGAATAATCGGATCTTCTTTAAGAATTTTAATTCGATTAGAATTAAGCC
10 PANJUG.G3.sample14_COI  100.0% 100.0%      AACTTTATATTTCTTATTTGGAATTTGAGCAGGAATAATCGGATCTTCTTTAAGAATTTTAATTCGATTAGAATTAAGCC
11 PANJUG.G3.sample15_COI  100.0% 100.0%      AACTTTATATTTCTTATTTGGAATTTGAGCAGGAATAATCGGATCTTCTTTAAGAATTTTAATTCGATTAGAATTAAGCC
12 PANJUG.G3.sample16_COI  100.0% 100.0%      AACTTTATATTTCTTATTTGGAATTTGAGCAGGAATAATCGGATCTTCTTTAAGAATTTTAATTCGATTAGAATTAAGCC
13 PANJUG.G3.sample17_COI  100.0% 100.0%      AACTTTATATTTCTTATTTGGAATTTGAGCAGGAATAATCGGATCTTCTTTAAGAATTTTAATTCGATTAGAATTAAGCC
14 PANJUG.G3.sample18_COI  100.0%  99.7%      AACATTATATTTCTTATTTGGAATTTGAGCAGGAATAATCGGATCTTCTTTAAGAATTTTAATTCGATTAGAATTAAGCC
15 PANJUG.G3.sample19_COI  100.0%  99.7%      AACATTATATTTCTTATTTGGAATTTGAGCAGGAATAATCGGATCTTCTTTAAGAATTTTAATTCGATTAGAATTAAGCC
16 PANJUG.G3.sample1_COI   100.0% 100.0%      AACTTTATATTTCTTATTTGGAATTTGAGCAGGAATAATCGGATCTTCTTTAAGAATTTTAATTCGATTAGAATTAAGCC
17 PANJUG.G3.sample20_COI  100.0%  99.8%      AACTTTATATTTCTTATTTGGAATTTGAGCAGGAATAATCGGATCTTCTTTAAGAATTTTAATTCGATTAGAATTAAGCC
18 PANJUG.G3.sample21_COI  100.0% 100.0%      AACTTTATATTTCTTATTTGGAATTTGAGCAGGAATAATCGGATCTTCTTTAAGAATTTTAATTCGATTAGAATTAAGCC
19 PANJUG.G3.sample22_COI  100.0% 100.0%      AACTTTATATTTCTTATTTGGAATTTGAGCAGGAATAATCGGATCTTCTTTAAGAATTTTAATTCGATTAGAATTAAGCC
20 PANJUG.G3.sample2_COI   100.0% 100.0%      AACTTTATATTTCTTATTTGGAATTTGAGCAGGAATAATCGGATCTTCTTTAAGAATTTTAATTCGATTAGAATTAAGCC
21 PANJUG.G3.sample3_COI   100.0% 100.0%      AACTTTATATTTCTTATTTGGAATTTGAGCAGGAATAATCGGATCTTCTTTAAGAATTTTAATTCGATTAGAATTAAGCC
22 PANJUG.G3.sample4_COI   100.0%  99.7%      AACATTATATTTCTTATTTGGAATTTGAGCAGGAATAATCGGATCTTCTTTAAGAATTTTAATTCGATTAGAATTAAGCC
23 PANJUG.G3.sample5_COI   100.0%  99.7%      AACATTATATTTCTTATTTGGAATTTGAGCAGGAATAATCGGATCTTCTTTAAGAATTTTAATTCGATTAGAATTAAGCC
24 PANJUG.G3.sample6_COI   100.0%  99.8%      AACTTTATATTTCTTATTTGGAATTTGAGCAGGAATAATCGGATCTTCTTTAAGAATTTTAATTCGATTAGAATTAAGCC
25 PANJUG.G3.sample7_COI   100.0% 100.0%      AACTTTATATTTCTTATTTGGAATTTGAGCAGGAATAATCGGATCTTCTTTAAGAATTTTAATTCGATTAGAATTAAGCC
26 PANJUG.G3.sample8_COI   100.0% 100.0%      AACTTTATATTTCTTATTTGGAATTTGAGCAGGAATAATCGGATCTTCTTTAAGAATTTTAATTCGATTAGAATTAAGCC
27 PANJUG.G3.sample9_COI   100.0% 100.0%      AACTTTATATTTCTTATTTGGAATTTGAGCAGGAATAATCGGATCTTCTTTAAGAATTTTAATTCGATTAGAATTAAGCC
```

```
                                 cov     pid  81         .         1         .         .         .         .         :         . 160
 1 PANJUG.G1.sample1_COI   100.0% 100.0%      AAATTAATTCAATTATTAATAATAACCAATTATATAATGTAATTGTAACAATTCATGCTTTTATTATAATTTTTTTTATA
 2 PANJUG.G1.sample2_COI   100.0% 100.0%      AAATTAATTCAATTATTAATAATAACCAATTATATAATGTAATTGTAACAATTCATGCTTTTATTATAATTTTTTTTATA
 3 PANJUG.G2.sample1_COI   100.0%  99.7%      AAATTAATTCAATTATTAATAATAACCAATTATATAATGTAATTGTAACAATTCATGCTTTTATTATAATTTTTTTTATA
 4 PANJUG.G2.sample2_COI   100.0%  99.7%      AAATTAATTCAATTATTAATAATAACCAATTATATAATGTAATTGTAACAATTCATGCTTTTATTATAATTTTTTTTATA
 5 PANJUG.G2.sample3_COI   100.0%  99.7%      AAATTAATTCAATTATTAATAATAACCAATTATATAATGTAATTGTAACAATTCATGCTTTTATTATAATTTTTTTTATA
 6 PANJUG.G3.sample10_COI  100.0% 100.0%      AAATTAATTCAATTATTAATAATAACCAATTATATAATGTAATTGTAACAATTCATGCTTTTATTATAATTTTTTTTATA
 7 PANJUG.G3.sample11_COI  100.0%  99.7%      AAATTAATTCAATTATTAATAATAACCAATTATATAATGTAATTGTAACAATTCATGCTTTTATTATAATTTTTTTTATA
 8 PANJUG.G3.sample12_COI  100.0%  99.7%      AAATTAATTCAATTATTAATAATAACCAATTATATAATGTAATTGTAACAATTCATGCTTTTATTATAATTTTTTTTATA
 9 PANJUG.G3.sample13_COI  100.0%  99.8%      AAATTAATTCAATTATTAATAATAACCAATTATATAATGTAATTGTAACAATTCATGCTTTTATTATAATTTTTTTTATA
10 PANJUG.G3.sample14_COI  100.0% 100.0%      AAATTAATTCAATTATTAATAATAACCAATTATATAATGTAATTGTAACAATTCATGCTTTTATTATAATTTTTTTTATA
```

11	PANJUG.G3.sample15_COI	100.0%	100.0%	AAATTAATTCAATTATTAATAATAACCAATTATATAATGTAATTGTAACAATTCATGCTTTTATTATAATTTTTTTTATA
12	PANJUG.G3.sample16_COI	100.0%	100.0%	AAATTAATTCAATTATTAATAATAACCAATTATATAATGTAATTGTAACAATTCATGCTTTTATTATAATTTTTTTTATA
13	PANJUG.G3.sample17_COI	100.0%	100.0%	AAATTAATTCAATTATTAATAATAACCAATTATATAATGTAATTGTAACAATTCATGCTTTTATTATAATTTTTTTTATA
14	PANJUG.G3.sample18_COI	100.0%	99.7%	AAATTAATTCAATTATTAATAATAACCAATTATATAATGTAATTGTAACAATTCATGCTTTTATTATAATTTTTTTTATA
15	PANJUG.G3.sample19_COI	100.0%	99.7%	AAATTAATTCAATTATTAATAATAACCAATTATATAATGTAATTGTAACAATTCATGCTTTTATTATAATTTTTTTTATA
16	PANJUG.G3.sample1_COI	100.0%	100.0%	AAATTAATTCAATTATTAATAATAACCAATTATATAATGTAATTGTAACAATTCATGCTTTTATTATAATTTTTTTTATA
17	PANJUG.G3.sample20_COI	100.0%	99.8%	AAATTAATTCAATTATTAATAATAACCAATTATATAATGTAATTGTAACAATTCATGCTTTTATTATAATTTTTTTTATA
18	PANJUG.G3.sample21_COI	100.0%	100.0%	AAATTAATTCAATTATTAATAATAACCAATTATATAATGTAATTGTAACAATTCATGCTTTTATTATAATTTTTTTTATA
19	PANJUG.G3.sample22_COI	100.0%	100.0%	AAATTAATTCAATTATTAATAATAACCAATTATATAATGTAATTGTAACAATTCATGCTTTTATTATAATTTTTTTTATA
20	PANJUG.G3.sample2_COI	100.0%	100.0%	AAATTAATTCAATTATTAATAATAACCAATTATATAATGTAATTGTAACAATTCATGCTTTTATTATAATTTTTTTTATA
21	PANJUG.G3.sample3_COI	100.0%	100.0%	AAATTAATTCAATTATTAATAATAACCAATTATATAATGTAATTGTAACAATTCATGCTTTTATTATAATTTTTTTTATA
22	PANJUG.G3.sample4_COI	100.0%	99.7%	AAATTAATTCAATTATTAATAATAACCAATTATATAATGTAATTGTAACAATTCATGCTTTTATTATAATTTTTTTTATA
23	PANJUG.G3.sample5_COI	100.0%	99.7%	AAATTAATTCAATTATTAATAATAACCAATTATATAATGTAATTGTAACAATTCATGCTTTTATTATAATTTTTTTTATA
24	PANJUG.G3.sample6_COI	100.0%	99.8%	AAATTAATTCAATTATTAATAATAACCAATTATATAATGTAATTGTAACAATTCATGCTTTTATTATAATTTTTTTTATA
25	PANJUG.G3.sample7_COI	100.0%	100.0%	AAATTAATTCAATTATTAATAATAACCAATTATATAATGTAATTGTAACAATTCATGCTTTTATTATAATTTTTTTTATA
26	PANJUG.G3.sample8_COI	100.0%	100.0%	AAATTAATTCAATTATTAATAATAACCAATTATATAATGTAATTGTAACAATTCATGCTTTTATTATAATTTTTTTTATA
27	PANJUG.G3.sample9_COI	100.0%	100.0%	AAATTAATTCAATTATTAATAATAACCAATTATATAATGTAATTGTAACAATTCATGCTTTTATTATAATTTTTTTTATA

		cov	pid	161 . . . 2 240
1	PANJUG.G1.sample1_COI	100.0%	100.0%	ACTATACCTATTGTAATTGGAGGGTTTGGAAATTGATTAATTCCATTAATAATAGGATGTCCAGATATATCATTTCCACG
2	PANJUG.G1.sample2_COI	100.0%	100.0%	ACTATACCTATTGTAATTGGAGGGTTTGGAAATTGATTAATTCCATTAATAATAGGATGTCCAGATATATCATTTCCACG
3	PANJUG.G2.sample1_COI	100.0%	99.7%	ACTATACCTATTGTAATTGGAGGGTTTGGAAATTGATTAATTCCATTAATAATAGGATGTCCAGATATATCATTTCCACG
4	PANJUG.G2.sample2_COI	100.0%	99.7%	ACTATACCTATTGTAATTGGAGGGTTTGGAAATTGATTAATTCCATTAATAATAGGATGTCCAGATATATCATTTCCACG
5	PANJUG.G2.sample3_COI	100.0%	99.7%	ACTATACCTATTGTAATTGGAGGGTTTGGAAATTGATTAATTCCATTAATAATAGGATGTCCAGATATATCATTTCCACG
6	PANJUG.G3.sample10_COI	100.0%	100.0%	ACTATACCTATTGTAATTGGAGGGTTTGGAAATTGATTAATTCCATTAATAATAGGATGTCCAGATATATCATTTCCACG
7	PANJUG.G3.sample11_COI	100.0%	99.7%	ACTATACCTATTGTAATTGGAGGGTTTGGAAATTGATTAATTCCATTAATAATAGGATGTCCAGATATATCATTTCCACG
8	PANJUG.G3.sample12_COI	100.0%	99.7%	ACTATACCTATTGTAATTGGAGGGTTTGGAAATTGATTAATTCCATTAATAATAGGATGTCCAGATATATCATTTCCACG
9	PANJUG.G3.sample13_COI	100.0%	99.8%	ACTATACCTATTGTAATTGGAGGGTTTGGAAATTGATTAATTCCATTAATAATAGGATGTCCAGATATATCATTTCCACG
10	PANJUG.G3.sample14_COI	100.0%	100.0%	ACTATACCTATTGTAATTGGAGGGTTTGGAAATTGATTAATTCCATTAATAATAGGATGTCCAGATATATCATTTCCACG
11	PANJUG.G3.sample15_COI	100.0%	100.0%	ACTATACCTATTGTAATTGGAGGGTTTGGAAATTGATTAATTCCATTAATAATAGGATGTCCAGATATATCATTTCCACG
12	PANJUG.G3.sample16_COI	100.0%	100.0%	ACTATACCTATTGTAATTGGAGGGTTTGGAAATTGATTAATTCCATTAATAATAGGATGTCCAGATATATCATTTCCACG
13	PANJUG.G3.sample17_COI	100.0%	100.0%	ACTATACCTATTGTAATTGGAGGGTTTGGAAATTGATTAATTCCATTAATAATAGGATGTCCAGATATATCATTTCCACG
14	PANJUG.G3.sample18_COI	100.0%	99.7%	ACTATACCTATTGTAATTGGAGGGTTTGGAAATTGATTAATTCCATTAATAATAGGATGTCCAGATATATCATTTCCACG
15	PANJUG.G3.sample19_COI	100.0%	99.7%	ACTATACCTATTGTAATTGGAGGGTTTGGAAATTGATTAATTCCATTAATAATAGGATGTCCAGATATATCATTTCCACG
16	PANJUG.G3.sample1_COI	100.0%	100.0%	ACTATACCTATTGTAATTGGAGGGTTTGGAAATTGATTAATTCCATTAATAATAGGATGTCCAGATATATCATTTCCACG
17	PANJUG.G3.sample20_COI	100.0%	99.8%	ACTATACCTATTGTAATTGGAGGGTTTGGAAATTGATTAATTCCATTAATAATAGGATGTCCAGATATATCATTTCCACG
18	PANJUG.G3.sample21_COI	100.0%	100.0%	ACTATACCTATTGTAATTGGAGGGTTTGGAAATTGATTAATTCCATTAATAATAGGATGTCCAGATATATCATTTCCACG
19	PANJUG.G3.sample22_COI	100.0%	100.0%	ACTATACCTATTGTAATTGGAGGGTTTGGAAATTGATTAATTCCATTAATAATAGGATGTCCAGATATATCATTTCCACG
20	PANJUG.G3.sample2_COI	100.0%	100.0%	ACTATACCTATTGTAATTGGAGGGTTTGGAAATTGATTAATTCCATTAATAATAGGATGTCCAGATATATCATTTCCACG
21	PANJUG.G3.sample3_COI	100.0%	100.0%	ACTATACCTATTGTAATTGGAGGGTTTGGAAATTGATTAATTCCATTAATAATAGGATGTCCAGATATATCATTTCCACG
22	PANJUG.G3.sample4_COI	100.0%	99.7%	ACTATACCTATTGTAATTGGAGGGTTTGGAAATTGATTAATTCCATTAATAATAGGATGTCCAGATATATCATTTCCACG
23	PANJUG.G3.sample5_COI	100.0%	99.7%	ACTATACCTATTGTAATTGGAGGGTTTGGAAATTGATTAATTCCATTAATAATAGGATGTCCAGATATATCATTTCCACG
24	PANJUG.G3.sample6_COI	100.0%	99.8%	ACTATACCTATTGTAATTGGAGGGTTTGGAAATTGATTAATTCCATTAATAATAGGATGTCCAGATATATCATTTCCACG
25	PANJUG.G3.sample7_COI	100.0%	100.0%	ACTATACCTATTGTAATTGGAGGGTTTGGAAATTGATTAATTCCATTAATAATAGGATGTCCAGATATATCATTTCCACG
26	PANJUG.G3.sample8_COI	100.0%	100.0%	ACTATACCTATTGTAATTGGAGGGTTTGGAAATTGATTAATTCCATTAATAATAGGATGTCCAGATATATCATTTCCACG

27	PANJUG.G3.sample9_COI	100.0%	100.0%	ACTATACCTATTGTAATTGGAGGGTTTGGAAATTGATTAATTCCATTAATAATAGGATGTCCAGATATATCATTTCCACG

		cov	pid	241 : . . . . 3 . . 320
1	PANJUG.G1.sample1_COI	100.0%	100.0%	ATTAAATAATATTAGATTTTGACTTTTACCTCCATCTTTAATAATAATAATTTCTAGATTTTTAATTAATAATGGTACAG
2	PANJUG.G1.sample2_COI	100.0%	100.0%	ATTAAATAATATTAGATTTTGACTTTTACCTCCATCTTTAATAATAATAATTTCTAGATTTTTAATTAATAATGGTACAG
3	PANJUG.G2.sample1_COI	100.0%	99.7%	ATTAAATAATATTAGATTTTGACTTTTACCTCCATCTTTAATAATAATAATTTCTAGATTTTTAATTAATAATGGTACAG
4	PANJUG.G2.sample2_COI	100.0%	99.7%	ATTAAATAATATTAGATTTTGACTTTTACCTCCATCTTTAATAATAATAATTTCTAGATTTTTAATTAATAATGGTACAG
5	PANJUG.G2.sample3_COI	100.0%	99.7%	ATTAAATAATATTAGATTTTGACTTTTACCTCCATCTTTAATAATAATAATTTCTAGATTTTTAATTAATAATGGTACAG
6	PANJUG.G3.sample10_COI	100.0%	100.0%	ATTAAATAATATTAGATTTTGACTTTTACCTCCATCTTTAATAATAATAATTTCTAGATTTTTAATTAATAATGGTACAG
7	PANJUG.G3.sample11_COI	100.0%	99.7%	ATTAAATAATATTAGATTTTGACTTTTACCTCCATCTTTAATAATAATAATTTCTAGATTTTTAATTAATAATGGTACAG
8	PANJUG.G3.sample12_COI	100.0%	99.7%	ATTAAATAATATTAGATTTTGACTTTTACCTCCATCTTTAATAATAATAATTTCTAGATTTTTAATTAATAATGGTACAG
9	PANJUG.G3.sample13_COI	100.0%	99.8%	ATTAAATAATATTAGATTTTGACTTTTACCTCCATCTTTAATAATAATAATTTCTAGATTTTTAATTAATAATGGTACAG
10	PANJUG.G3.sample14_COI	100.0%	100.0%	ATTAAATAATATTAGATTTTGACTTTTACCTCCATCTTTAATAATAATAATTTCTAGATTTTTAATTAATAATGGTACAG
11	PANJUG.G3.sample15_COI	100.0%	100.0%	ATTAAATAATATTAGATTTTGACTTTTACCTCCATCTTTAATAATAATAATTTCTAGATTTTTAATTAATAATGGTACAG
12	PANJUG.G3.sample16_COI	100.0%	100.0%	ATTAAATAATATTAGATTTTGACTTTTACCTCCATCTTTAATAATAATAATTTCTAGATTTTTAATTAATAATGGTACAG
13	PANJUG.G3.sample17_COI	100.0%	100.0%	ATTAAATAATATTAGATTTTGACTTTTACCTCCATCTTTAATAATAATAATTTCTAGATTTTTAATTAATAATGGTACAG
14	PANJUG.G3.sample18_COI	100.0%	99.7%	ATTAAATAATATTAGATTTTGACTTTTACCTCCATCTTTAATAATAATAATTTCTAGATTTTTAATTAATAATGGTACAG
15	PANJUG.G3.sample19_COI	100.0%	99.7%	ATTAAATAATATTAGATTTTGACTTTTACCTCCATCTTTAATAATAATAATTTCTAGATTTTTAATTAATAATGGTACAG
16	PANJUG.G3.sample1_COI	100.0%	100.0%	ATTAAATAATATTAGATTTTGACTTTTACCTCCATCTTTAATAATAATAATTTCTAGATTTTTAATTAATAATGGTACAG
17	PANJUG.G3.sample20_COI	100.0%	99.8%	ATTAAATAATATTAGATTTTGACTTTTACCTCCATCTTTAATAATAATAATTTCTAGATTTTTAATTAATAATGGTACAG
18	PANJUG.G3.sample21_COI	100.0%	100.0%	ATTAAATAATATTAGATTTTGACTTTTACCTCCATCTTTAATAATAATAATTTCTAGATTTTTAATTAATAATGGTACAG
19	PANJUG.G3.sample22_COI	100.0%	100.0%	ATTAAATAATATTAGATTTTGACTTTTACCTCCATCTTTAATAATAATAATTTCTAGATTTTTAATTAATAATGGTACAG
20	PANJUG.G3.sample2_COI	100.0%	100.0%	ATTAAATAATATTAGATTTTGACTTTTACCTCCATCTTTAATAATAATAATTTCTAGATTTTTAATTAATAATGGTACAG
21	PANJUG.G3.sample3_COI	100.0%	100.0%	ATTAAATAATATTAGATTTTGACTTTTACCTCCATCTTTAATAATAATAATTTCTAGATTTTTAATTAATAATGGTACAG
22	PANJUG.G3.sample4_COI	100.0%	99.7%	ATTAAATAATATTAGATTTTGACTTTTACCTCCATCTTTAATAATAATAATTTCTAGATTTTTAATTAATAATGGTACAG
23	PANJUG.G3.sample5_COI	100.0%	99.7%	ATTAAATAATATTAGATTTTGACTTTTACCTCCATCTTTAATAATAATAATTTCTAGATTTTTAATTAATAATGGTACAG
24	PANJUG.G3.sample6_COI	100.0%	99.8%	ATTAAATAATATTAGATTTTGACTTTTACCTCCATCTTTAATAATAATAATTTCTAGATTTTTAATTAATAATGGTACAG
25	PANJUG.G3.sample7_COI	100.0%	100.0%	ATTAAATAATATTAGATTTTGACTTTTACCTCCATCTTTAATAATAATAATTTCTAGATTTTTAATTAATAATGGTACAG
26	PANJUG.G3.sample8_COI	100.0%	100.0%	ATTAAATAATATTAGATTTTGACTTTTACCTCCATCTTTAATAATAATAATTTCTAGATTTTTAATTAATAATGGTACAG
27	PANJUG.G3.sample9_COI	100.0%	100.0%	ATTAAATAATATTAGATTTTGACTTTTACCTCCATCTTTAATAATAATAATTTCTAGATTTTTAATTAATAATGGTACAG

		cov	pid	321 . . : 4 400
1	PANJUG.G1.sample1_COI	100.0%	100.0%	GAACAGGTTGAACAATTTATCCACCTCTCTCAAATAACATTGCACATAATAATATTTCAGTTGATTTAACAATTTTCTCA
2	PANJUG.G1.sample2_COI	100.0%	100.0%	GAACAGGTTGAACAATTTATCCACCTCTCTCAAATAACATTGCACATAATAATATTTCAGTTGATTTAACAATTTTCTCA
3	PANJUG.G2.sample1_COI	100.0%	99.7%	GAACAGGTTGAACAATTTATCCACCTCTCTCAAATAACATTGCACATAATAATATTTCAGTTGATTTAACAATTTTCTCA
4	PANJUG.G2.sample2_COI	100.0%	99.7%	GAACAGGTTGAACAATTTATCCACCTCTCTCAAATAACATTGCACATAATAATATTTCAGTTGATTTAACAATTTTCTCA
5	PANJUG.G2.sample3_COI	100.0%	99.7%	GAACAGGTTGAACAATTTATCCACCTCTCTCAAATAACATTGCACATAATAATATTTCAGTTGATTTAACAATTTTCTCA
6	PANJUG.G3.sample10_COI	100.0%	100.0%	GAACAGGTTGAACAATTTATCCACCTCTCTCAAATAACATTGCACATAATAATATTTCAGTTGATTTAACAATTTTCTCA
7	PANJUG.G3.sample11_COI	100.0%	99.7%	GAACAGGTTGAACAATTTATCCACCTCTCTCAAATAACATTGCACATAATAATATTTCAGTTGATTTAACAATTTTCTCA
8	PANJUG.G3.sample12_COI	100.0%	99.7%	GAACAGGTTGAACAATTTATCCACCTCTCTCAAATAACATTGCACATAATAATATTTCAGTTGATTTAACAATTTTCTCA
9	PANJUG.G3.sample13_COI	100.0%	99.8%	GAACAGGTTGAACAATTTATCCACCTCTCTCAAATAACATTGCACATAATAATATTTCAGTTGATTTAACAATTTTCTCA
10	PANJUG.G3.sample14_COI	100.0%	100.0%	GAACAGGTTGAACAATTTATCCACCTCTCTCAAATAACATTGCACATAATAATATTTCAGTTGATTTAACAATTTTCTCA
11	PANJUG.G3.sample15_COI	100.0%	100.0%	GAACAGGTTGAACAATTTATCCACCTCTCTCAAATAACATTGCACATAATAATATTTCAGTTGATTTAACAATTTTCTCA
12	PANJUG.G3.sample16_COI	100.0%	100.0%	GAACAGGTTGAACAATTTATCCACCTCTCTCAAATAACATTGCACATAATAATATTTCAGTTGATTTAACAATTTTCTCA

```
13 PANJUG.G3.sample17_COI 100.0% 100.0%   GAACAGGTTGAACAATTTATCCACCTCTCTCAAATAACATTGCACATAATAATATTTCAGTTGATTTAACAATTTTCTCA
14 PANJUG.G3.sample18_COI 100.0%  99.7%   GAACAGGTTGAACAATTTATCCACCTCTCTCAAATAACATTGCACATAATAATATTTCAGTTGATTTAACAATTTTCTCA
15 PANJUG.G3.sample19_COI 100.0%  99.7%   GAACAGGTTGAACAATTTATCCACCTCTCTCAAATAACATTGCACATAATAATATTTCAGTTGATTTAACAATTTTCTCA
16 PANJUG.G3.sample1_COI  100.0% 100.0%   GAACAGGTTGAACAATTTATCCACCTCTCTCAAATAACATTGCACATAATAATATTTCAGTTGATTTAACAATTTTCTCA
17 PANJUG.G3.sample20_COI 100.0%  99.8%   GAACAGGTTGAACAATTTATCCACCTCTCTCAAATAACATTGCACATAATAATATTTCAGTTGATTTAACAATTTTCTCA
18 PANJUG.G3.sample21_COI 100.0% 100.0%   GAACAGGTTGAACAATTTATCCACCTCTCTCAAATAACATTGCACATAATAATATTTCAGTTGATTTAACAATTTTCTCA
19 PANJUG.G3.sample22_COI 100.0% 100.0%   GAACAGGTTGAACAATTTATCCACCTCTCTCAAATAACATTGCACATAATAATATTTCAGTTGATTTAACAATTTTCTCA
20 PANJUG.G3.sample2_COI  100.0% 100.0%   GAACAGGTTGAACAATTTATCCACCTCTCTCAAATAACATTGCACATAATAATATTTCAGTTGATTTAACAATTTTCTCA
21 PANJUG.G3.sample3_COI  100.0% 100.0%   GAACAGGTTGAACAATTTATCCACCTCTCTCAAATAACATTGCACATAATAATATTTCAGTTGATTTAACAATTTTCTCA
22 PANJUG.G3.sample4_COI  100.0%  99.7%   GAACAGGTTGAACAATTTATCCACCTCTCTCAAATAACATTGCACATAATAATATTTCAGTTGATTTAACAATTTTCTCA
23 PANJUG.G3.sample5_COI  100.0%  99.7%   GAACAGGTTGAACAATTTATCCACCTCTCTCAAATAACATTGCACATAATAATATTTCAGTTGATTTAACAATTTTCTCA
24 PANJUG.G3.sample6_COI  100.0%  99.8%   GAACAGGTTGAACAATTTATCCACCTCTCTCAAATAACATTGCACATAATAATATTTCAGTTGATTTAACAATTTTCTCA
25 PANJUG.G3.sample7_COI  100.0% 100.0%   GAACAGGTTGAACAATTTATCCACCTCTCTCAAATAACATTGCACATAATAATATTTCAGTTGATTTAACAATTTTCTCA
26 PANJUG.G3.sample8_COI  100.0% 100.0%   GAACAGGTTGAACAATTTATCCACCTCTCTCAAATAACATTGCACATAATAATATTTCAGTTGATTTAACAATTTTCTCA
27 PANJUG.G3.sample9_COI  100.0% 100.0%   GAACAGGTTGAACAATTTATCCACCTCTCTCAAATAACATTGCACATAATAATATTTCAGTTGATTTAACAATTTTCTCA

                            cov    pid 401          .         .         .         .         :         .         .         . 480
 1 PANJUG.G1.sample1_COI  100.0% 100.0%   TTACACTTAGCAGGAATCTCATCAATTTTAGGAGCTATTAATTTTATTTGTACAATTCTTAATATAATACCAAATAATTT
 2 PANJUG.G1.sample2_COI  100.0% 100.0%   TTACACTTAGCAGGAATCTCATCAATTTTAGGAGCTATTAATTTTATTTGTACAATTCTTAATATAATACCAAATAATTT
 3 PANJUG.G2.sample1_COI  100.0%  99.7%   TTACACTTAGCAGGAATCTCATCAATTTTAGGAGCTATTAATTTTATTTGTACAATTCTTAATATAATACCAAATAATTT
 4 PANJUG.G2.sample2_COI  100.0%  99.7%   TTACACTTAGCAGGAATCTCATCAATTTTAGGAGCTATTAATTTTATTTGTACAATTCTTAATATAATACCAAATAATTT
 5 PANJUG.G2.sample3_COI  100.0%  99.7%   TTACACTTAGCAGGAATCTCATCAATTTTAGGAGCTATTAATTTTATTTGTACAATTCTTAATATAATACCAAATAATTT
 6 PANJUG.G3.sample10_COI 100.0% 100.0%   TTACACTTAGCAGGAATCTCATCAATTTTAGGAGCTATTAATTTTATTTGTACAATTCTTAATATAATACCAAATAATTT
 7 PANJUG.G3.sample11_COI 100.0%  99.7%   TTACACTTAGCAGGAATCTCATCAATTTTAGGAGCTATTAATTTTATTTGTACAATTCTTAATATAATACCAAATAATTT
 8 PANJUG.G3.sample12_COI 100.0%  99.7%   TTACACTTAGCAGGAATCTCATCAATTTTAGGAGCTATTAATTTTATTTGTACAATTCTTAATATAATACCAAATAATTT
 9 PANJUG.G3.sample13_COI 100.0%  99.8%   TTACACTTAGCAGGAATCTCATCAATTTTAGGAGCTATTAATTTTATTTGTACAATTCTTAATATAATACCAAATAATTT
10 PANJUG.G3.sample14_COI 100.0% 100.0%   TTACACTTAGCAGGAATCTCATCAATTTTAGGAGCTATTAATTTTATTTGTACAATTCTTAATATAATACCAAATAATTT
11 PANJUG.G3.sample15_COI 100.0% 100.0%   TTACACTTAGCAGGAATCTCATCAATTTTAGGAGCTATTAATTTTATTTGTACAATTCTTAATATAATACCAAATAATTT
12 PANJUG.G3.sample16_COI 100.0% 100.0%   TTACACTTAGCAGGAATCTCATCAATTTTAGGAGCTATTAATTTTATTTGTACAATTCTTAATATAATACCAAATAATTT
13 PANJUG.G3.sample17_COI 100.0% 100.0%   TTACACTTAGCAGGAATCTCATCAATTTTAGGAGCTATTAATTTTATTTGTACAATTCTTAATATAATACCAAATAATTT
14 PANJUG.G3.sample18_COI 100.0%  99.7%   TTACACTTAGCAGGAATCTCATCAATTTTAGGAGCTATTAATTTTATTTGTACAATTCTTAATATAATACCAAATAATTT
15 PANJUG.G3.sample19_COI 100.0%  99.7%   TTACACTTAGCAGGAATCTCATCAATTTTAGGAGCTATTAATTTTATTTGTACAATTCTTAATATAATACCAAATAATTT
16 PANJUG.G3.sample1_COI  100.0% 100.0%   TTACACTTAGCAGGAATCTCATCAATTTTAGGAGCTATTAATTTTATTTGTACAATTCTTAATATAATACCAAATAATTT
17 PANJUG.G3.sample20_COI 100.0%  99.8%   TTACACTTAGCAGGAATCTCATCAATTTTAGGAGCTATTAATTTTATTTGTACAATTCTTAATATAATACCAAATAATTT
18 PANJUG.G3.sample21_COI 100.0% 100.0%   TTACACTTAGCAGGAATCTCATCAATTTTAGGAGCTATTAATTTTATTTGTACAATTCTTAATATAATACCAAATAATTT
19 PANJUG.G3.sample22_COI 100.0% 100.0%   TTACACTTAGCAGGAATCTCATCAATTTTAGGAGCTATTAATTTTATTTGTACAATTCTTAATATAATACCAAATAATTT
20 PANJUG.G3.sample2_COI  100.0% 100.0%   TTACACTTAGCAGGAATCTCATCAATTTTAGGAGCTATTAATTTTATTTGTACAATTCTTAATATAATACCAAATAATTT
21 PANJUG.G3.sample3_COI  100.0% 100.0%   TTACACTTAGCAGGAATCTCATCAATTTTAGGAGCTATTAATTTTATTTGTACAATTCTTAATATAATACCAAATAATTT
22 PANJUG.G3.sample4_COI  100.0%  99.7%   TTACACTTAGCAGGAATCTCATCAATTTTAGGAGCTATTAATTTTATTTGTACAATTCTTAATATAATACCAAATAATTT
23 PANJUG.G3.sample5_COI  100.0%  99.7%   TTACACTTAGCAGGAATCTCATCAATTTTAGGAGCTATTAATTTTATTTGTACAATTCTTAATATAATACCAAATAATTT
24 PANJUG.G3.sample6_COI  100.0%  99.8%   TTACACTTAGCAGGAATCTCATCAATTTTAGGAGCTATTAATTTTATTTGTACAATTCTTAATATAATACCAAATAATTT
25 PANJUG.G3.sample7_COI  100.0% 100.0%   TTACACTTAGCAGGAATCTCATCAATTTTAGGAGCTATTAATTTTATTTGTACAATTCTTAATATAATACCAAATAATTT
26 PANJUG.G3.sample8_COI  100.0% 100.0%   TTACACTTAGCAGGAATCTCATCAATTTTAGGAGCTATTAATTTTATTTGTACAATTCTTAATATAATACCAAATAATTT
27 PANJUG.G3.sample9_COI  100.0% 100.0%   TTACACTTAGCAGGAATCTCATCAATTTTAGGAGCTATTAATTTTATTTGTACAATTCTTAATATAATACCAAATAATTT
```

```
                                        cov     pid 481         .         5         .         .         .         .         :         . 560
 1 PANJUG.G1.sample1_COI   100.0% 100.0%     AAAATTAAATCAAATTCCATTATTTCCCTGATCAATTTTAATTACTGCTATTTTACTAATTATTTCACTACCAGTTTTAG
 2 PANJUG.G1.sample2_COI   100.0% 100.0%     AAAATTAAATCAAATTCCATTATTTCCCTGATCAATTTTAATTACTGCTATTTTACTAATTATTTCACTACCAGTTTTAG
 3 PANJUG.G2.sample1_COI   100.0%  99.7%     AAAATTAAATCAAATTCCATTATTTCCCTGATCAATTTTAATTACTGCTATTTTACTAATTATTTCACTACCAGTTTTAG
 4 PANJUG.G2.sample2_COI   100.0%  99.7%     AAAATTAAATCAAATTCCATTATTTCCCTGATCAATTTTAATTACTGCTATTTTACTAATTATTTCACTACCAGTTTTAG
 5 PANJUG.G2.sample3_COI   100.0%  99.7%     AAAATTAAATCAAATTCCATTATTTCCCTGATCAATTTTAATTACTGCTATTTTACTAATTATTTCACTACCAGTTTTAG
 6 PANJUG.G3.sample10_COI  100.0% 100.0%     AAAATTAAATCAAATTCCATTATTTCCCTGATCAATTTTAATTACTGCTATTTTACTAATTATTTCACTACCAGTTTTAG
 7 PANJUG.G3.sample11_COI  100.0%  99.7%     AAAATTAAATCAAATTCCATTATTTCCCTGATCAATTTTAATTACTGCTATTTTACTAATTATTTCACTACCAGTTTTAG
 8 PANJUG.G3.sample12_COI  100.0%  99.7%     AAAATTAAATCAAATTCCATTATTTCCCTGATCAATTTTAATTACTGCTATTTTACTAATTATTTCACTACCAGTTTTAG
 9 PANJUG.G3.sample13_COI  100.0%  99.8%     AAAATTAAATCAAATTCCATTATTTCCCTGATCAATTTTAATTACTGCTATTTTACTAATTATTTCACTACCAGTTTTAG
10 PANJUG.G3.sample14_COI  100.0% 100.0%     AAAATTAAATCAAATTCCATTATTTCCCTGATCAATTTTAATTACTGCTATTTTACTAATTATTTCACTACCAGTTTTAG
11 PANJUG.G3.sample15_COI  100.0% 100.0%     AAAATTAAATCAAATTCCATTATTTCCCTGATCAATTTTAATTACTGCTATTTTACTAATTATTTCACTACCAGTTTTAG
12 PANJUG.G3.sample16_COI  100.0% 100.0%     AAAATTAAATCAAATTCCATTATTTCCCTGATCAATTTTAATTACTGCTATTTTACTAATTATTTCACTACCAGTTTTAG
13 PANJUG.G3.sample17_COI  100.0% 100.0%     AAAATTAAATCAAATTCCATTATTTCCCTGATCAATTTTAATTACTGCTATTTTACTAATTATTTCACTACCAGTTTTAG
14 PANJUG.G3.sample18_COI  100.0%  99.7%     AAAATTAAATCAAATTCCATTATTTCCCTGATCAATTTTAATTACTGCTATTTTACTAATTATTTCACTACCAGTTTTAG
15 PANJUG.G3.sample19_COI  100.0%  99.7%     AAAATTAAATCAAATTCCATTATTTCCCTGATCAATTTTAATTACTGCTATTTTACTAATTATTTCACTACCAGTTTTAG
16 PANJUG.G3.sample1_COI   100.0% 100.0%     AAAATTAAATCAAATTCCATTATTTCCCTGATCAATTTTAATTACTGCTATTTTACTAATTATTTCACTACCAGTTTTAG
17 PANJUG.G3.sample20_COI  100.0%  99.8%     AAAATTAAATCAAATTCCATTATTTCCCTGATCAATTTTAATTACTGCTATTTTACTAATTATTTCACTACCAGTTTTAG
18 PANJUG.G3.sample21_COI  100.0% 100.0%     AAAATTAAATCAAATTCCATTATTTCCCTGATCAATTTTAATTACTGCTATTTTACTAATTATTTCACTACCAGTTTTAG
19 PANJUG.G3.sample22_COI  100.0% 100.0%     AAAATTAAATCAAATTCCATTATTTCCCTGATCAATTTTAATTACTGCTATTTTACTAATTATTTCACTACCAGTTTTAG
20 PANJUG.G3.sample2_COI   100.0% 100.0%     AAAATTAAATCAAATTCCATTATTTCCCTGATCAATTTTAATTACTGCTATTTTACTAATTATTTCACTACCAGTTTTAG
21 PANJUG.G3.sample3_COI   100.0% 100.0%     AAAATTAAATCAAATTCCATTATTTCCCTGATCAATTTTAATTACTGCTATTTTACTAATTATTTCACTACCAGTTTTAG
22 PANJUG.G3.sample4_COI   100.0%  99.7%     AAAATTAAATCAAATTCCATTATTTCCCTGATCAATTTTAATTACTGCTATTTTACTAATTATTTCACTACCAGTTTTAG
23 PANJUG.G3.sample5_COI   100.0%  99.7%     AAAATTAAATCAAATTCCATTATTTCCCTGATCAATTTTAATTACTGCTATTTTACTAATTATTTCACTACCAGTTTTAG
24 PANJUG.G3.sample6_COI   100.0%  99.8%     AAAATTAAATCAAATTCCATTATTTCCCTGATCAATTTTAATTACTGCTATTTTACTAATTATTTCACTACCAGTTTTAG
25 PANJUG.G3.sample7_COI   100.0% 100.0%     AAAATTAAATCAAATTCCATTATTTCCCTGATCAATTTTAATTACTGCTATTTTACTAATTATTTCACTACCAGTTTTAG
26 PANJUG.G3.sample8_COI   100.0% 100.0%     AAAATTAAATCAAATTCCATTATTTCCCTGATCAATTTTAATTACTGCTATTTTACTAATTATTTCACTACCAGTTTTAG
27 PANJUG.G3.sample9_COI   100.0% 100.0%     AAAATTAAATCAAATTCCATTATTTCCCTGATCAATTTTAATTACTGCTATTTTACTAATTATTTCACTACCAGTTTTAG

                                        cov     pid 561         .         .         .         6         .         .         .         . 640
 1 PANJUG.G1.sample1_COI   100.0% 100.0%     CTGGTGCCATTACAATACTATTAACTGACCGAAACCTAAATACATCTTTTTTTGATCCATCAGGAGGAGGTGATCCAATT
 2 PANJUG.G1.sample2_COI   100.0% 100.0%     CTGGTGCCATTACAATACTATTAACTGACCGAAACCTAAATACATCTTTTTTTGATCCATCAGGAGGAGGTGATCCAATT
 3 PANJUG.G2.sample1_COI   100.0%  99.7%     CTGGTGCCATTACAATACTATTAACTGACCGAAACCTAAATACATCTTTTTTTGATCCATCAGGAGGAGGTGATCCAATT
 4 PANJUG.G2.sample2_COI   100.0%  99.7%     CTGGTGCCATTACAATACTATTAACTGACCGAAACCTAAATACATCTTTTTTTGATCCATCAGGAGGAGGTGATCCAATT
 5 PANJUG.G2.sample3_COI   100.0%  99.7%     CTGGTGCCATTACAATACTATTAACTGACCGAAACCTAAATACATCTTTTTTTGATCCATCAGGAGGAGGTGATCCAATT
 6 PANJUG.G3.sample10_COI  100.0% 100.0%     CTGGTGCCATTACAATACTATTAACTGACCGAAACCTAAATACATCTTTTTTTGATCCATCAGGAGGAGGTGATCCAATT
 7 PANJUG.G3.sample11_COI  100.0%  99.7%     CTGGTGCCATTACAATACTATTAACTGACCGAAACCTAAATACATCTTTTTTTGATCCATCAGGAGGAGGTGATCCAATT
 8 PANJUG.G3.sample12_COI  100.0%  99.7%     CTGGTGCCATTACAATACTATTAACTGACCGAAACCTAAATACATCTTTTTTTGATCCATCAGGAGGAGGTGATCCAATT
 9 PANJUG.G3.sample13_COI  100.0%  99.8%     CTGGTGCCATTACAATACTATTAACTGACCGAAACCTAAATACATCTTTTTTTGATCCATCAGGAGGAGGTGATCCAATT
10 PANJUG.G3.sample14_COI  100.0% 100.0%     CTGGTGCCATTACAATACTATTAACTGACCGAAACCTAAATACATCTTTTTTTGATCCATCAGGAGGAGGTGATCCAATT
11 PANJUG.G3.sample15_COI  100.0% 100.0%     CTGGTGCCATTACAATACTATTAACTGACCGAAACCTAAATACATCTTTTTTTGATCCATCAGGAGGAGGTGATCCAATT
12 PANJUG.G3.sample16_COI  100.0% 100.0%     CTGGTGCCATTACAATACTATTAACTGACCGAAACCTAAATACATCTTTTTTTGATCCATCAGGAGGAGGTGATCCAATT
13 PANJUG.G3.sample17_COI  100.0% 100.0%     CTGGTGCCATTACAATACTATTAACTGACCGAAACCTAAATACATCTTTTTTTGATCCATCAGGAGGAGGTGATCCAATT
```

```
14 PANJUG.G3.sample18_COI 100.0%  99.7%   CTGGTGCCATTACAATACTATTAACTGACCGAAACCTAAATACATCTTTTTTTGATCCATCAGGAGGAGGTGATCCAATT
15 PANJUG.G3.sample19_COI 100.0%  99.7%   CTGGTGCCATTACAATACTATTAACTGACCGAAACCTAAATACATCTTTTTTTGATCCATCAGGAGGAGGTGATCCAATT
16 PANJUG.G3.sample1_COI  100.0% 100.0%   CTGGTGCCATTACAATACTATTAACTGACCGAAACCTAAATACATCTTTTTTTGATCCATCAGGAGGAGGTGATCCAATT
17 PANJUG.G3.sample20_COI 100.0%  99.8%   CTGGTGCCATTACAATACTATTAACTGACCGAAACCTAAATACATCTTTTTTTGATCCATCAGGAGGAGGTGATCCAATT
18 PANJUG.G3.sample21_COI 100.0% 100.0%   CTGGTGCCATTACAATACTATTAACTGACCGAAACCTAAATACATCTTTTTTTGATCCATCAGGAGGAGGTGATCCAATT
19 PANJUG.G3.sample22_COI 100.0% 100.0%   CTGGTGCCATTACAATACTATTAACTGACCGAAACCTAAATACATCTTTTTTTGATCCATCAGGAGGAGGTGATCCAATT
20 PANJUG.G3.sample2_COI  100.0% 100.0%   CTGGTGCCATTACAATACTATTAACTGACCGAAACCTAAATACATCTTTTTTTGATCCATCAGGAGGAGGTGATCCAATT
21 PANJUG.G3.sample3_COI  100.0% 100.0%   CTGGTGCCATTACAATACTATTAACTGACCGAAACCTAAATACATCTTTTTTTGATCCATCAGGAGGAGGTGATCCAATT
22 PANJUG.G3.sample4_COI  100.0%  99.7%   CTGGTGCCATTACAATACTATTAACTGACCGAAACCTAAATACATCTTTTTTTGATCCATCAGGAGGAGGTGATCCAATT
23 PANJUG.G3.sample5_COI  100.0%  99.7%   CTGGTGCCATTACAATACTATTAACTGACCGAAACCTAAATACATCTTTTTTTGATCCATCAGGAGGAGGTGATCCAATT
24 PANJUG.G3.sample6_COI  100.0%  99.8%   CTGGTGCCATTACAATACTATTAACTGACCGAAACCTAAATACATCTTTTTTTGATCCATCAGGAGGAGGTGATCCAATT
25 PANJUG.G3.sample7_COI  100.0% 100.0%   CTGGTGCCATTACAATACTATTAACTGACCGAAACCTAAATACATCTTTTTTTGATCCATCAGGAGGAGGTGATCCAATT
26 PANJUG.G3.sample8_COI  100.0% 100.0%   CTGGTGCCATTACAATACTATTAACTGACCGAAACCTAAATACATCTTTTTTTGATCCATCAGGAGGAGGTGATCCAATT
27 PANJUG.G3.sample9_COI  100.0% 100.0%   CTGGTGCCATTACAATACTATTAACTGACCGAAACCTAAATACATCTTTTTTTGATCCATCAGGAGGAGGTGATCCAATT

                             cov    pid 641           :      ] 658
 1 PANJUG.G1.sample1_COI  100.0% 100.0%   TTATATCAACATTTATTC
 2 PANJUG.G1.sample2_COI  100.0% 100.0%   TTATATCAACATTTATTC
 3 PANJUG.G2.sample1_COI  100.0%  99.7%   TTATATCAACATTTATTT
 4 PANJUG.G2.sample2_COI  100.0%  99.7%   TTATATCAACATTTATTT
 5 PANJUG.G2.sample3_COI  100.0%  99.7%   TTATATCAACATTTATTT
 6 PANJUG.G3.sample10_COI 100.0% 100.0%   TTATATCAACATTTATTC
 7 PANJUG.G3.sample11_COI 100.0%  99.7%   TTATATCAACATTTATTT
 8 PANJUG.G3.sample12_COI 100.0%  99.7%   TTATATCAACATTTATTT
 9 PANJUG.G3.sample13_COI 100.0%  99.8%   TTATATCAACATTTATTT
10 PANJUG.G3.sample14_COI 100.0% 100.0%   TTATATCAACATTTATTC
11 PANJUG.G3.sample15_COI 100.0% 100.0%   TTATATCAACATTTATTC
12 PANJUG.G3.sample16_COI 100.0% 100.0%   TTATATCAACATTTATTC
13 PANJUG.G3.sample17_COI 100.0% 100.0%   TTATATCAACATTTATTC
14 PANJUG.G3.sample18_COI 100.0%  99.7%   TTATATCAACATTTATTT
15 PANJUG.G3.sample19_COI 100.0%  99.7%   TTATATCAACATTTATTT
16 PANJUG.G3.sample1_COI  100.0% 100.0%   TTATATCAACATTTATTC
17 PANJUG.G3.sample20_COI 100.0%  99.8%   TTATATCAACATTTATTT
18 PANJUG.G3.sample21_COI 100.0% 100.0%   TTATATCAACATTTATTC
19 PANJUG.G3.sample22_COI 100.0% 100.0%   TTATATCAACATTTATTC
20 PANJUG.G3.sample2_COI  100.0% 100.0%   TTATATCAACATTTATTC
21 PANJUG.G3.sample3_COI  100.0% 100.0%   TTATATCAACATTTATTC
22 PANJUG.G3.sample4_COI  100.0%  99.7%   TTATATCAACATTTATTT
23 PANJUG.G3.sample5_COI  100.0%  99.7%   TTATATCAACATTTATTT
24 PANJUG.G3.sample6_COI  100.0%  99.8%   TTATATCAACATTTATTT
25 PANJUG.G3.sample7_COI  100.0% 100.0%   TTATATCAACATTTATTC
26 PANJUG.G3.sample8_COI  100.0% 100.0%   TTATATCAACATTTATTC
27 PANJUG.G3.sample9_COI  100.0% 100.0%   TTATATCAACATTTATTC
```
