# Supplementary material for: First Report on the Acrobat Ant Crematogaster scutellaris Storing Live Aphids in Its Oak-Gall Nests
Source: Insects. 2021 Jan 27;12(2):108. doi: 10.3390/insects12020108 (PMC7912212; doi:10.3390/insects12020108)
Supplement: Supplementary file 1 [file insects-12-00108-s001.zip › Table S3.pdf]

Supplementary file 3. Summary of the polymorphic sites in the *P. juglandis* COI sequences evidenced by the alignment and identification of the three observed haplotypes (in black, red and green).

```
PANJUG.G1.sample1_COI    AACTTTATATTTCTTATTTGGAATTTGAGCAGGAATAATCGGATCTTCTT
PANJUG.G1.sample2_COI    AACTTTATATTTCTTATTTGGAATTTGAGCAGGAATAATCGGATCTTCTT
PANJUG.G2.sample1_COI    AACATTATATTTCTTATTTGGAATTTGAGCAGGAATAATCGGATCTTCTT
PANJUG.G2.sample2_COI    AACATTATATTTCTTATTTGGAATTTGAGCAGGAATAATCGGATCTTCTT
PANJUG.G2.sample3_COI    AACATTATATTTCTTATTTGGAATTTGAGCAGGAATAATCGGATCTTCTT
PANJUG.G3.sample10_COI   AACTTTATATTTCTTATTTGGAATTTGAGCAGGAATAATCGGATCTTCTT
PANJUG.G3.sample11_COI   AACATTATATTTCTTATTTGGAATTTGAGCAGGAATAATCGGATCTTCTT
PANJUG.G3.sample12_COI   AACATTATATTTCTTATTTGGAATTTGAGCAGGAATAATCGGATCTTCTT
PANJUG.G3.sample13_COI   AACTTTATATTTCTTATTTGGAATTTGAGCAGGAATAATCGGATCTTCTT
PANJUG.G3.sample14_COI   AACTTTATATTTCTTATTTGGAATTTGAGCAGGAATAATCGGATCTTCTT
PANJUG.G3.sample15_COI   AACTTTATATTTCTTATTTGGAATTTGAGCAGGAATAATCGGATCTTCTT
PANJUG.G3.sample16_COI   AACTTTATATTTCTTATTTGGAATTTGAGCAGGAATAATCGGATCTTCTT
PANJUG.G3.sample17_COI   AACTTTATATTTCTTATTTGGAATTTGAGCAGGAATAATCGGATCTTCTT
PANJUG.G3.sample18_COI   AACATTATATTTCTTATTTGGAATTTGAGCAGGAATAATCGGATCTTCTT
PANJUG.G3.sample19_COI   AACATTATATTTCTTATTTGGAATTTGAGCAGGAATAATCGGATCTTCTT
PANJUG.G3.sample1_COI    AACTTTATATTTCTTATTTGGAATTTGAGCAGGAATAATCGGATCTTCTT
PANJUG.G3.sample20_COI   AACTTTATATTTCTTATTTGGAATTTGAGCAGGAATAATCGGATCTTCTT
PANJUG.G3.sample21_COI   AACTTTATATTTCTTATTTGGAATTTGAGCAGGAATAATCGGATCTTCTT
PANJUG.G3.sample22_COI   AACTTTATATTTCTTATTTGGAATTTGAGCAGGAATAATCGGATCTTCTT
PANJUG.G3.sample2_COI    AACTTTATATTTCTTATTTGGAATTTGAGCAGGAATAATCGGATCTTCTT
PANJUG.G3.sample3_COI    AACTTTATATTTCTTATTTGGAATTTGAGCAGGAATAATCGGATCTTCTT
PANJUG.G3.sample4_COI    AACATTATATTTCTTATTTGGAATTTGAGCAGGAATAATCGGATCTTCTT
PANJUG.G3.sample5_COI    AACATTATATTTCTTATTTGGAATTTGAGCAGGAATAATCGGATCTTCTT
PANJUG.G3.sample6_COI    AACTTTATATTTCTTATTTGGAATTTGAGCAGGAATAATCGGATCTTCTT
PANJUG.G3.sample7_COI    AACTTTATATTTCTTATTTGGAATTTGAGCAGGAATAATCGGATCTTCTT
PANJUG.G3.sample8_COI    AACTTTATATTTCTTATTTGGAATTTGAGCAGGAATAATCGGATCTTCTT
PANJUG.G3.sample9_COI    AACTTTATATTTCTTATTTGGAATTTGAGCAGGAATAATCGGATCTTCTT
                         *** **********************************************
```

(....)

```
PANJUG.G1.sample1_COI    ATTTATTC
PANJUG.G1.sample2_COI    ATTTATTC
PANJUG.G2.sample1_COI    ATTTATTT
PANJUG.G2.sample2_COI    ATTTATTT
PANJUG.G2.sample3_COI    ATTTATTT
PANJUG.G3.sample10_COI   ATTTATTC
PANJUG.G3.sample11_COI   ATTTATTT
PANJUG.G3.sample12_COI   ATTTATTT
PANJUG.G3.sample13_COI   ATTTATTT
PANJUG.G3.sample14_COI   ATTTATTC
PANJUG.G3.sample15_COI   ATTTATTC
PANJUG.G3.sample16_COI   ATTTATTC
PANJUG.G3.sample17_COI   ATTTATTC
PANJUG.G3.sample18_COI   ATTTATTT
PANJUG.G3.sample19_COI   ATTTATTT
PANJUG.G3.sample1_COI    ATTTATTC
PANJUG.G3.sample20_COI   ATTTATTT
PANJUG.G3.sample21_COI   ATTTATTC
PANJUG.G3.sample22_COI   ATTTATTC
PANJUG.G3.sample2_COI    ATTTATTC
PANJUG.G3.sample3_COI    ATTTATTC
PANJUG.G3.sample4_COI    ATTTATTT
PANJUG.G3.sample5_COI    ATTTATTT
PANJUG.G3.sample6_COI    ATTTATTT
PANJUG.G3.sample7_COI    ATTTATTC
PANJUG.G3.sample8_COI    ATTTATTC
PANJUG.G3.sample9_COI    ATTTATTC
```

****
